# A Novel Biclustering Algorithm for the Discovery of Meaningful Biological Correlations between microRNAs and their Target Genes

**DOI:** 10.1186/1471-2105-14-S7-S8

**Published:** 2013-04-22

**Authors:** Gianvito Pio, Michelangelo Ceci, Domenica D'Elia, Corrado Loglisci, Donato Malerba

**Affiliations:** 1 Department of Computer Science, University of Bari "Aldo Moro", Via Orabona 4, 70125, Bari, Italy; 2 CNR, Institute for Biomedical Technologies, Via Amendola 122/D, 70126, Bari, Italy

## Abstract

**Background:**

microRNAs (miRNAs) are a class of small non-coding RNAs which have been recognized as ubiquitous post-transcriptional regulators. The analysis of interactions between different miRNAs and their target genes is necessary for the understanding of miRNAs' role in the control of cell life and death. In this paper we propose a novel data mining algorithm, called HOCCLUS2, specifically designed to bicluster miRNAs and target messenger RNAs (mRNAs) on the basis of their experimentally-verified and/or predicted interactions. Indeed, existing biclustering approaches, typically used to analyze gene expression data, fail when applied to miRNA:mRNA interactions since they usually do not extract possibly overlapping biclusters (miRNAs and their target genes may have multiple roles), extract a huge amount of biclusters (difficult to browse and rank on the basis of their importance) and work on similarities of feature values (do not limit the analysis to reliable interactions).

**Results:**

To overcome these limitations, HOCCLUS2 *i) *extracts possibly overlapping biclusters, to catch multiple roles of both miRNAs and their target genes; *ii) *extracts hierarchically organized biclusters, to facilitate bicluster browsing and to distinguish between universe and pathway-specific miRNAs; *iii) *extracts highly cohesive biclusters, to consider only reliable interactions; *iv) *ranks biclusters according to the functional similarities, computed on the basis of Gene Ontology, to facilitate bicluster analysis.

**Conclusions:**

Our results show that HOCCLUS2 is a valid tool to support biologists in the identification of context-specific miRNAs regulatory modules and in the detection of possibly unknown miRNAs target genes. Indeed, results prove that HOCCLUS2 is able to extract cohesiveness-preserving biclusters, when compared with competitive approaches, and statistically confirm (at a confidence level of 99%) that mRNAs which belong to the same biclusters are, on average, more functionally similar than mRNAs which belong to different biclusters. Finally, the hierarchy of biclusters provides useful insights to understand the intrinsic hierarchical organization of miRNAs and their potential multiple interactions on target genes.

## Background

miRNAs are post-transcriptional regulators which represent one of the major regulatory gene families in animals, plants and viruses. They bind to complementary sequences on target mRNAs, resulting in negative regulation (transcript degradation and sequestering, translational suppression) or positive regulation (transcriptional and translational activation) [1,2]. Studies on interactions between miRNAs and their target genes are of the utmost importance to understand the role of miRNAs in controlling cell processes and metabolic pathways [3] as well as to discover unknown functional synergies.

This work aims to contribute to the elucidation of miRNAs' complex biological functions by proposing a method for biclustering miRNAs and mRNAs. Biclustering is a data mining task whose goal, similar to the classical clustering, is to group together similar objects. The difference is that objects that fall in the same cluster are of two different types. Furthermore, objects of one type are clustered together according to their relationships with objects of the other type (symmetrically).

The method we propose identifies (possibly unknown) highly connected networks of miRNAs and mRNAs, that is, regulatory networks/modules. Thus, the aim is to provide the biologists with a tool which can support them in two challenging tasks: the identification of context-specific miRNAs regulatory modules and the detection of (possibly previously unknown) miRNAs target genes.

As recognized in [4], the problem of discovering regulatory modules that control gene transcription in biological model systems can be solved by applying biclustering algorithms. Consequently, several papers in the literature apply biclustering in the biological domain [5-9]. However, they work on gene expression data and not on miRNA:mRNA interactions. In order to work properly on miRNA:mRNA interactions, some important issues have to be considered. In particular, extracted biclusters should be:

• Possibly overlapping, since mRNAs and miRNAs can be involved in multiple regulatory networks [10]. Ignoring this aspect would lead to the identification of incomplete interaction networks.

• Hierarchically organized. This organization facilitates the interpretation of results, even when a high number of biclusters is extracted. Moreover, it opens the opportunity to consider an intrinsic hierarchical organization of miRNAs, where it is possible to distinguish between miRNAs involved in many signaling pathways (universe miRNAs) and pathway-specific miRNAs (intra-pathway miRNAs). The latter aspect has recently been considered an important issue that deserves deeper investigation [11].

• Highly cohesive. This means that miRNAs and mRNAs in the same bicluster should be highly related and show (only) reliable interactions. This is different from what biclustering methods specifically designed for gene expression data do, that is, grouping together genes and conditions with similar (both high and low) expression values.

We propose an algorithm for the efficient discovery of overlapping, hierarchically organized and highly cohesive biclusters. Biclusters are extracted from a dataset of experimentally verified miRNA:mRNA interactions, i.e. miRTarBase [12], as well as from miRNAs target prediction datasets extracted from mirDIP [11]. In the latter case, the integration of different miRNA target prediction algorithms contributes to reducing the impact of noise (i.e., false positives) on the significance of the resulting biclusters.

Besides the extraction and evaluation of potential regulatory modules (expressed as biclusters), this paper provides a way to systematically assess the actual role of miRNAs in biclusters in the control of biological processes [3] in which their target mRNAs are involved. This analysis is performed by exploiting a statistical significance test, whose goal is to evaluate the hypothesis that mRNAs which belong to the same biclusters are, on average, more functionally similar than mRNAs which belong to different biclusters. In this test, the functional similarity is evaluated according to the Gene Ontology (GO) [13] classification.

Furthermore, we provide a ranking of biclusters on the basis of an additional statistical test which compares intra-and inter-functional similarity of *each *bicluster with respect to the GO classification. This ranking aims to simplify the identification of the most significant biclusters.

### Related works

The research reported in this paper has its roots in works which study biclustering (/co-clustering) algorithms for biological data mining, as well as in works which study the role of miRNA:mRNA regulatory modules. Regarding the first research line, we only concentrate on algorithms which extract overlapping biclusters, since in our context, as previously stated, extracting non-overlapping biclusters is too limitative.

#### Extraction of overlapping biclusters for biological data analysis

**Figure 1:**
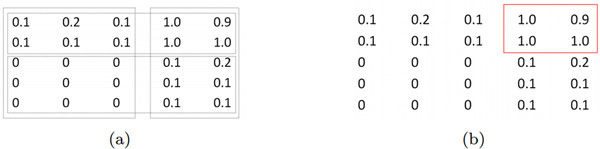
**Similarity-based vs. cohesiveness-based biclustering approaches**. (a) Biclustering obtained by means of (most of) similarity-based approaches. (b) Biclustering obtained by means of cohesiveness-based approaches.

There are several papers in the literature that deal with the extraction of overlapping biclusters. Most of them are applied or specifically designed for gene expression data analysis. In this setting, gene expression data are organized as matrices/tables, where rows represent genes, columns represent various samples such as tissues or experimental conditions, and values in each cell characterize the expression level of the particular gene in the particular sample. According to this setting, biclustering methods typically group together rows (columns) with similar (both high and low) expression values, which, as previously stated, is different from our goal of maximizing the cohesiveness (see Figure 1). In the following, we describe these methods.

One of the pioneering works on this topic [8] proposes a greedy heuristic search to generate arbitrarily positioned, overlapping biclusters, based on a homogeneity constraint. In this case, biclustering is based on iterative insertions and deletions of genes and conditions asymmetrically (i.e. insertions and deletions of conditions depend on insertions and deletions of genes). Since biclustering is guided by only one dimension, rows and columns are not interchangeable. Moreover, as pointed out in [9], this iterative algorithm is computationally expensive, since it identifies individual biclusters sequentially rather than all at once. The algorithm also causes random perturbations to the data since it inserts random values instead of deleting rows and columns corresponding to the previously discovered bicluster. This process, although allowing overlapping, can reduce the biclustering quality.

In [14], the authors propose initializing (possibly overlapping) biclusters with random rows and columns and, then, iteratively moving rows/columns among them. Each "move" operation aims to minimize the *mean residue *which indicates the degree of coherence of a cell value with the remaining values in the bicluster. This approach has the disadvantage that it is significantly dependent on the initial random biclustering. As in [8], this approach is not deterministic and does not extract hierarchically organized biclusters. Contrary to [8], it discovers biclusters all at once, thus improving computational efficiency.

A different solution is proposed in [7], where genes and conditions are represented according to a binary matrix, which is recursively divided into two smaller (possibly overlapping) submatrices, after a rearrangement of columns/rows. Since re-arrangement is computationally expensive [15], the proposed solution is impractical for large datasets.

In [5], biclustering is guided by a probabilistic process by means of which objects are assigned to hierarchically organized clusters on a single dimension. Clustering on this dimension determines clustering on the other dimension (where it is possible to have overlapping). This means that the hierarchy is defined only on the first dimension and overlapping is supported only on the second dimension.

In [9], the authors propose the algorithm ROCC which rearranges columns and rows in order to identify the most coherent biclusters (expressed as submatrices). Subsequently, it works in a bottom-up fashion and iteratively merges pairs of "closest" biclusters until a stopping criterion is satisfied. ROCC bases the merging process on "relationships" (i.e. submatrices) and not directly on objects (i.e. rows and columns), with the consequence that it may encounter problems when processing datasets affected by "relational" imbalance (i.e. when objects of different types participate with significantly different cardinalities in the interactions) [16]. Although this algorithm is, in principle, able to extract a hierarchy of biclusters, it only returns the set of biclusters obtained at the last iteration.

In the literature there have been a few attempts to work on miRNA:mRNA interactions [6,10,17]. These works will be introduced and described in the next subsection.

#### miRNA:mRNA regulatory modules

Several works in the literature have studied different facets of the interactions among miRNAs, genes and proteins. In particular: [1], [18] and [19] study the global miRNA regulation in cellular networks; [20] and [21] study the combinatorial miRNA regulation in cellular pathways; [22] and [23] study the correspondence between regulatory networks extracted from transcriptional and miRNA data; [24] studies and proves that miRNAs tend to target highly connected genes or proteins in cellular networks; [11] combines multiple miRNA prediction databases to identify signaling pathway-associated miRNAs.

However, approaches for full-scale analysis of the regulatory networks spanned by miRNAs are only now getting under way [10]. These approaches have their roots in studies which, aiming to identify a modular organization of biological networks (see, for instance [25]), have pointed out that such networks have greatly advanced our understanding of complex cellular systems. As recognized in [10], identifying functional miRNA:mRNA regulatory modules is a challenging task for several reasons: (i) one mRNA can be regulated by multiple miRNAs and one miRNA can regulate a large number of mRNAs. (ii) miRNA:mRNA specific interactions often differ in a cell-type and cell-phase dependent manner. (iii) although miRNAs physically interact with mRNAs, ultimately miRNA regulation affects the quantity of proteins in cells rather than the quantity of mRNAs. Thus, the expression levels of miRNAs are not always exactly anti-correlated with those of their target genes. While (i) and (ii) motivate the use of biclustering approaches which extract overlapping biclusters, (iii) suggests the use of miRNA target predictions (in alternative to the experimentally verified interactions) extracted by appropriate algorithms.

Following this stream of research, in [6] the authors have proposed an algorithm to identify miRNA:mRNA regulatory modules based on predicted miRNA:mRNA target information. This algorithm extracts maximal bicliques (complete bipartite graphs) which represent candidate biclusters. From candidate biclusters, only those for which the range of scores of miRNA:gene interactions are in a user-defined interval are returned. Consequently, this algorithm suffers from the problem of manually setting the interval and from the problem that the extraction of bicliques prevents the algorithm from identifying non-completely connected interaction networks, which results in a high number of (redundant) small biclusters. Moreover, since this algorithm is based on a method specifically designed for gene expression data [26], it does not extract highly cohesive biclusters. Finally, extracted biclusters are not hierarchically organized. These limitations can also be found in [17], where the method is similar to that proposed in [6]. Here, however, the extraction of bicliques also takes into account coherent expression patterns between miRNAs and genes, or the (anti)-correlations between each miRNA-target gene pair.

In [10], the proposed solution aims to extract biclusters by solving a non-negative matrix factorization problem. The peculiarity of this approach is that it takes into account additional information coming from protein-protein interaction networks and from gene expression data. Also in this case, high cohesion is not guaranteed and extracted biclusters are not hierarchically organized.

### Contributions

Taking into account all the considerations reported so far, we propose an algorithm, called HOCCLUS2 (Hierarchical Overlapping Co-CLUStering2), which provides a solution to the issues raised by the specific task in hand and effectively deals with the "relational" imbalance problem. Moreover, it does not require as input the number of desired biclusters, i.e. it is able to automatically determine the optimal number of biclusters, by exploiting information about the underlying data distribution. The algorithm starts from an initial set of biclusters which express bicliques (fully connected bipartite graphs) and, then, iteratively defines the hierarchical organization of biclusters according to a bottom-up strategy.

This paper is based on the preliminary work in [16], where only the system HOCCLUS is presented. However, this paper significantly extends and upgrades the work presented there:

• We propose a novel algorithm for the construction of the initial biclusters which are now expressed as overlapping bicliques. This is different from what is done in HOCCLUS, where the system METIS [27] is adapted to extract (non-overlapping) biclusters. This difference is crucial, since METIS can extract biclusters that do not represent fully connected subgraphs. Consequently, it is possible that very specific biclusters are lost. Moreover, biclusters discovered by METIS depend on a user-defined parameter: the number of initial biclusters. Manual tuning of this parameter is an open problem in METIS.

• We revise the method in order to consider the possible presence of "noise" objects. This is coherent with the basic principle of some well-established and well-known clustering algorithms such as DBSCAN [28].

• We report a theoretical analysis of the time complexity of the learning algorithm.

• We report an extended experimental analysis of experimentally verified miRNA:mRNA interactions and miRNA target prediction datasets (miRTarBase and mirDIP, respectively). This is different from [16], where the analysis is only performed on miRNAMap 2.0 [29].

• We use statistical tests to evaluate the hypothesis that mRNAs which belong to the same biclusters are more functionally similar (according to GO) than mRNAs which belong to different biclusters.

• We provide a ranking of biclusters on the basis of a statistical analysis.

## Methods

The method we propose is based on three main steps:

**1** Extraction of a set of initial non-hierarchically organized biclusters.

**2** An iterative process in which, at each iteration, two phases are performed, that is, overlap identification and merging. In the former, some objects (miRNAs or mRNAs) belonging to a bicluster can be added to another bicluster. In the latter, biclusters are merged when some heuristic criteria are satisfied. It is noteworthy that at each iteration several pairs of biclusters can be merged. Moreover, at each iteration, depending on whether merging is performed, an additional level of the hierarchy may or may not be added. This process stops when neither overlaps nor merges are performed in the last iteration.

**3** A ranking of extracted biclusters. Ranking takes into account a preference function which exploits the intra-and inter-functional similarities (according to GO) of objects in each bicluster.

It is noteworthy that the iterative merging process (Step 2) can be applied to biclusters consisting of a single miRNA:mRNA interaction. Although this solution would make Step 1 useless, it would lead to the construction of a very large set of meaningless hierarchy levels. The construction of an initial set of biclusters guarantees the significance of the results, even from the first level of the hierarchy.

Before formally introducing the problem we intend to solve, some useful definitions are necessary: let *V_r _*and *V_c _*be the sets of mRNAs and miRNAs, respectively (subscripts *r *and *c *stand for row and column, respectively. Here rows refer to mRNAs and columns refer to miRNAs. They are actually interchangeable in HOCCLUS2). Let *A^n × m ^*be an adjacency matrix, where *n *= |*V_r_*|, *m *= |*V_c_*| and *A_r_*_(*x*)_,*_c_*_(*y*) _is a score associated to the interaction between *x *∈ *V_r _*and *y *∈ *V_c_*, where *r *: *V_r _*→ [1, *n*](*c *: *V_c _*→ [1, *m*]) is a function that maps a row (column) object to the corresponding row (column) index of the matrix *A*. Without loss of generality, we impose that ∀*x *∈ *V_r_*, ∀*y *∈ *V_c _*: *A_r_*_(*x*),_*_c_*_(*y*) _∈ [0,1], where 0 means no interaction and 1 means the most reliable interaction.

Formally, the problem is defined as follows:

Given:

• the set of mRNAs *V_r_*, the set of miRNAs *V_c_*;

• the adjacency matrix *A^n×m^*;

• a minimum interaction score β;

• a cohesiveness function $q:C\times {\left[0,1\right]}^{n\;\;\times \;\;m}\to \mathbb{R}$$\left(C=(2^{V_{r}}\cup 2^{V_{c}}\backslash \{\emptyset \}\right)$ is the set of possible biclusters);

• a cohesiveness threshold *α *for $q\left(\cdot ,\cdot \right)$;

• a preference function p:C→ℝ.

*Find: *a ranked list of biclusters *L_j _*for each level *j *= 1, . . . , *k *such that:

a) for each list *L_j_*, *j *= 2, . . . , *k *we have that ∀ *C' *∈ *L_j _*∃ *C" *∈ *L_j_*_−1_, such that *C" *⊆ *C' *(hierarchy);

b) biclusters at the same level can share objects in *V_r _*and in *V_c _*(overlapping);

c) for each bicluster *C' *∈ *L*_1_; ∀*x *∈ *V_r _*∩ *C' *, ∀*y *∈ *V_c _*∩ *C' *, *A_r_*_(*x*),_*_c_*_(*y*) _> β;

d) for each bicluster *C' *∈ *L_j _*obtained after merging, *q*(*C' *, *A*) ≥ *α *(cohesiveness);

e) for each pair of biclusters *C' *, *C" *∈ *L_j _*, *p*(*C' *) ≥ *p*(*C" *) iff *C' *precedes *C" *in *L_j _*.

It is noteworthy that, at this stage, we do not impose additional conditions on the cohesiveness function *q*(·,·) and on the preference function *p*(·) which will be defined later. Moreover, *L_k _*does not necessarily contain a single bicluster, meaning that a forest of biclusters is actually returned. This is coherent with the task in hand, where some sets of miRNAs could be totally unrelated to some sets of mRNAs. Moreover, *α *implicitly influences the number *k *of the levels and the number of biclusters at each hierarchy level.

**Figure 2:**
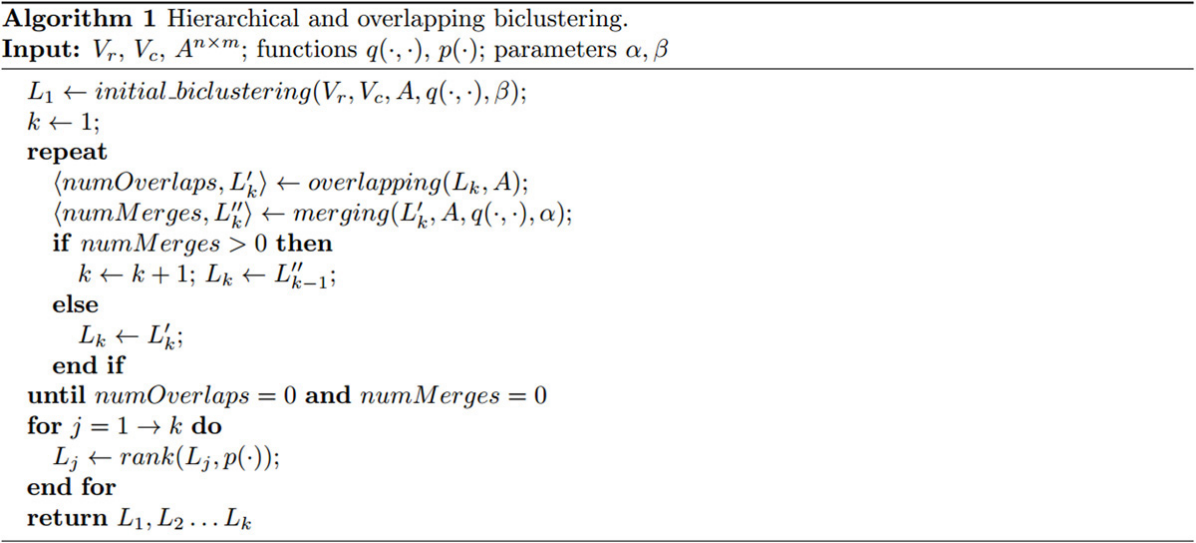
**Hierarchical and overlapping biclustering algorithm**. High level description (in pseudocode) of the proposed hierarchical and overlapping biclustering algorithm.

Algorithm reported in Figure 2 solves the considered problem. Single steps will be detailed in the following subsections.

### Building the initial biclusters

We consider two different alternatives for this task. The first one consists in exploiting an existing biclustering algorithm. For this purpose, we use the algorithm *METIS *[27]. *METIS *is a good candidate for working with miRNA:mRNA interactions, since it aims at minimizing the so-called edge-cut of the graph and, consequently, at maximizing the intra-cluster cohesiveness. METIS, although originally designed for classical clustering problems, can extract miRNA:mRNA biclusters by forcing node weights such that both miRNAs and mRNAs must appear in the same cluster (http://glaros.dtc.umn.edu/gkhome/node/685). However, METIS, as most of biclustering algorithms, requires as input the desired number of biclusters. Although in experiments this issue is not perceived, since they are often performed on real/synthetic datasets where the number of biclusters is already known, it is a relevant problem in real contexts, such as in the analysis of gene expression data or miRNA:mRNA interactions. Moreover, METIS is exhaustive, i.e. each object (miRNA and mRNA, in this case) is always assigned to a bicluster. This characteristic leads to low-quality biclusters when some mRNAs (resp. miRNAs) do not share with other mRNAs (resp. miRNAs) a significant number of strong interactions with miRNAs (resp. mRNAs). According to the considerations provided in [28], these objects can be considered as noise objects, since located in low-density areas of the space, and should be automatically discarded.

The second alternative consists in the use of a new algorithm which overcomes these limitations. The only parameter the proposed algorithm requires is *β *∈ [0,1], whose value can be easily chosen by experts, since it represents the minimum score for miRNA:mRNA interactions. Interactions with score values less than β are ignored, thus β implicitly defines a sort of filter on the reliability of the interactions.

The algorithm builds biclusters in the form of bicliques by analyzing interactions in two directions, i.e. from miRNA to mRNA and from mRNA to miRNA. Once a set of bicliques is obtained for each direction, they are merged together to obtain the final set of bicliques. Since the algorithm works in a symmetrical way, we here describe only the extraction of the initial bicliques in the "miRNA to mRNA" direction.

**Figure 3:**
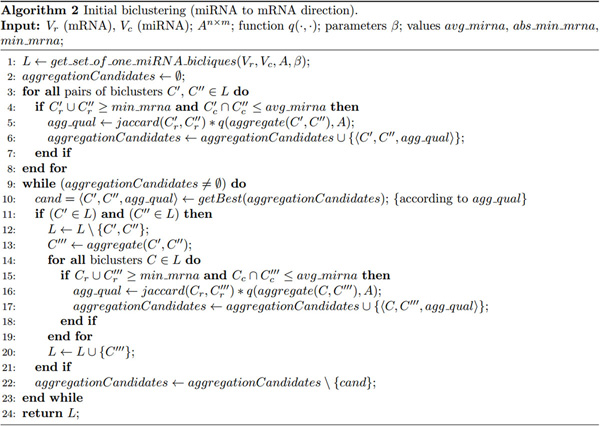
**Initial biclustering algorithm**. High level description (in pseudocode) of the initial biclustering algorithm (miRNA to mRNA direction).

The algorithm (see Figure 3) works by taking into account some statistical properties, that is:

• *avg_mirna: *the average number of miRNAs which target each mRNA, with a score greater than β;

• *abs_min_mrna *and *min_mrna*: the absolute and the *outlier-proof *(respectively) minimum number of mRNAs which are targeted by each miRNA, with a score greater than β.

The *min_mrna *value (*outlier-proof*) is computed by assuming that the number of mRNAs which are targeted by each miRNA follows a Normal distribution. In particular, we take the minimum number of targeted mRNAs (with a score greater then β) by discarding the lowest 0.15% values, which are possibly outliers, according to the 99.7 (or three-sigma) rule. Symmetrically, *avg_mrna*, *abs_min_mirna *and *min_mirna *are calculated for the "mRNA to miRNA" direction.

**Figure 4:**
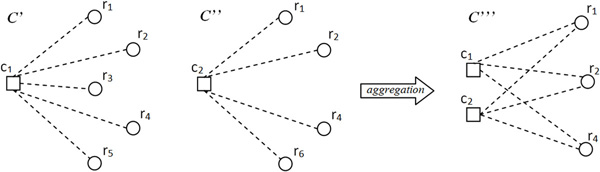
**Aggregation**. An example of aggregation of two bicliques (*C' *and *C"*) into a new biclique (*C'"*), according to Equation (1).

Once these simple statistics are computed, an initial set of bicliques is built. Each initial biclique consists of a single miRNA and the set of mRNAs it targets with a score greater than β, so that we have at most |*V_c_*| initial bicliques (Figure 3, line 1). The algorithm, then, iteratively aggregates two biclusters *C' *and *C" *into a new bicluster *C''' *as follows (see Figure 3, lines 9-23, and Figure 4):

(1) $$C_{r}^{\prime \prime \prime }=C_{r}^{\prime }\cap C_{r}^{\prime \prime }\;\;C_{c}^{\prime \prime \prime }=C_{c}^{\prime }\cup C_{c}^{\prime \prime }$$

where *C_r _*= *V_r _*∩ *C *and *C_c _*= *V_c _*∩ *C *represent row and column objects in *C*, respectively.

Aggregation is based on the property that the number of miRNAs is antimonotonic with respect to the number of mRNAs in a biclique. The necessary conditions for aggregating are (Figure 3, lines 4 and 15):

(2) $$C_{r}^{\prime }\cup C_{r}^{\prime \prime }\geq min\text{\_}mrna\;\;C_{c}^{\prime }\cap C_{c}^{\prime \prime }\leq avg\text{\_}mirna$$

The basic idea is that a good biclique should contain approximately *avg_mirna *miRNAs, while keeping the highest possible number of mRNAs (at least *min_mrna*). Moreover, as the goal of the algorithm is to obtain a set of highly cohesive bicliques, among the possible aggregations of pairs of bicliques 〈*C'*, *C"*〉 we select the one for which the following measure is maximized (Figure 3, line 10):

(3) $$jaccard\left(C_{r}^{\prime },C_{r}^{\prime \prime }\right)*q\left(aggregate\left(C\prime ,C^{\prime \prime }\right),A\right)$$

where $jaccard\left(C_{r}^{\prime },C_{r}^{\prime \prime }\;\right)=\frac{\left|{C^{\prime }}_{r}\cap {C^{\prime \prime }}_{r}\right|}{\left|{C^{\prime }}_{r}\cup {C^{\prime \prime }}_{r}\right|}$, *A *is the adjacency matrix and *q*(·,·) is a cohesiveness function. The cohesiveness function that we consider in this work is defined as follows:

(4) $$q\left(C,A\right)=\frac{\underset{x\in C_{r}}{\sum }\underset{y\in C_{c}}{\sum }A_{r\left(x\right),c\left(y\right)}}{\left|C_{r}|*|C_{c}\right|}$$

This function measures the weighted (i.e. by considering the score of the interactions) percentage of interactions in a bicluster, normalized by the maximum number of possible interactions. Intuitively, the function *q *measures the intra-cluster cohesion (also known as "compactness" in classical clustering).

The iterative process stops when there are no additional candidates for aggregation, i.e. there is no pair of biclusters which satisfies the conditions of the inequalities in (2).

The whole process is also performed in the "mRNA to miRNA" direction and the two sets of biclusters are then merged by simply removing biclusters which appear more than once and biclusters which are a subset of others. The algorithm then starts a pruning phase whose goal is to remove noise objects. Coherently with the definition of noise objects provided before, each bicluster containing less than *abs_min_mirna *miRNAs or less than *abs_min_mrna *mRNAs is removed. We recall that both *abs_min_mirna *and *abs_min_mrna *are computed according to a statistical analysis of the data.

### Overlap identification

The basic assumption behind the overlap identification is that two non-overlapping biclusters should be (linearly or not) separable in the space. According to this assumption, we identify objects belonging to one bicluster that can be added to another bicluster. In particular, given two biclusters *C' *and *C" *(belonging to the same level of the hierarchy), *C' *≠ *C"*, we identify two optimal separating hyperplanes between *C' *and *C" *by learning an SVM model for each dimension (miRNAs and mRNAs). Since our goal is not to build a good predictive classification model, but to evaluate the separability of objects belonging to different biclusters, the objects in *C' *and *C" *are used as both the training set and the testing set. Misclassified objects are those which possibly belong to both the considered biclusters. Intuitively, the separating hyperplane can be interpreted as delineating the changes of the underlying data distribution between *C' *and *C"*. This is coherent with studies that exploit SVMs for solving clustering tasks [30].

When learning SVMs, each row (column) object is represented as its corresponding row (column) vector of *A*. The use of SVMs as discriminative methods is motivated by their recognized peculiarity in dealing with sparse data [31], that is a common situation in a miRNAs:mRNAs adjacency matrix.

More formally, we build two binary classifiers: $SVM_{{C^{\prime }}_{r},{C^{\prime \prime }}_{r}}:{\left[0,1\right]}^{m}\to \left\{0,1\right\}$ and $SVM_{{C^{\prime }}_{c},{C^{\prime \prime }}_{c}}:{\left[0,1\right]}^{n}\to \left\{0,1\right\}$. Once the classifiers are built,

• for each row object $x\in \left(C_{r}^{\prime }\cup C_{r}^{\prime \prime }\right)$, we consider its corresponding row vector *A_r_*_(*x*),*_

- if $SVM_{{C^{\prime }}_{r},{C^{\prime \prime }}_{r}}\left(A_{r\left(x\right),*}\right)=0$ and $x\in C_{r}^{\prime \prime }$, then $C_{r}^{\prime }\leftarrow C_{r}^{\prime }\cup \left\{x\right\}$

- if $SVM_{{C^{\prime }}_{r},{C^{\prime \prime }}_{r}}\left(A_{r\left(x\right),*}\right)=1$ and $x\in C_{r}^{\prime }$, then $C_{r}^{\prime \prime }\leftarrow C_{r}^{\prime \prime }\cup \left\{x\right\}$

• for each column object $y\in \left(C_{c}^{\prime }\cup C_{c}^{\prime \prime }\right)$, we consider its corresponding column vector A_*,*c*(*y*)_

- if $SVM_{{C^{\prime }}_{c},{C^{\prime \prime }}_{c}}\left(A_{*,c\left(y\right)}\right)=0$ and $y\in C_{c}^{\prime \prime }$, then $C_{c}^{\prime }\leftarrow C_{c}^{\prime }\cup \left\{y\right\}$

- if $SVM_{{C^{\prime }}_{c},{C^{\prime \prime }}_{c}}\left(A_{*,c\left(y\right)}\right)=1$ and $y\in C_{c}^{\prime }$, then $C_{c}^{\prime \prime }\leftarrow C_{c}^{\prime \prime }\cup \left\{y\right\}$

**Figure 5:**
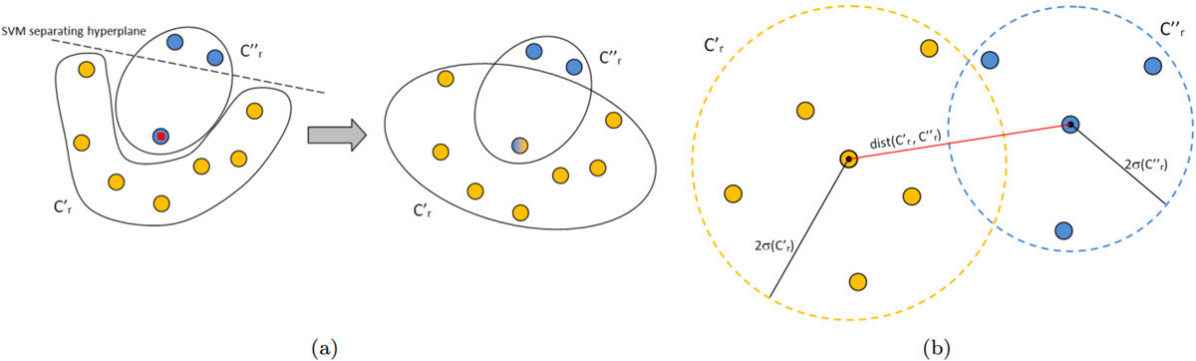
**Overlapping and merging phases**. (a) Overlapping between two clusters along one dimension. The red marked object (misclassified) is added to the other cluster. (b) An example of the object distribution of the row dimension of the biclusters *C' *and *C"*. In this case, *C' *and *C" *are candidates for merging.

In this way we obtain overlapping biclusters, where the common objects are those that cannot be correctly classified by the separating hyperplane (Figure 5(a)).

It is noteworthy that SVMs have to be constructed on each pair of biclusters for each level. In order to obtain a result which is independent of the order in which pairs of biclusters are analyzed, the misclassified objects are added at the end of the overlap identification process.

In Figure 2, *overlapping*(*L_k_*, *A*) is in charge of identifying possible overlaps. It returns the number of objects that have been added to biclusters and the updated set of biclusters with added objects. In our implementation, the algorithm used for learning SVMs is SMO [32] with the default kernel (linear).

### Merging

Once a set of overlapping biclusters has been obtained, we can analyze them to evaluate if some pairs of biclusters can be reasonably merged. A naïve approach would consider only the distance or the number of common objects, neglecting their statistical distribution. Here, we assume that row (column) objects in a bicluster are normally distributed in the space [0,1]*^m ^*([0,1]*^n^*), that is, in the space in which their row (column) vectors are represented. We consider the distance between pairs of biclusters in order to merge those for which a defined percentage of (possibly unknown) objects can statistically be in common.

Formally, two biclusters *C'*, *C" *are candidates for merging if:

(5) $$dist\left(C_{r}^{\prime },C_{r}^{\prime \prime }\right)-2\sigma \left(C_{r}^{\prime }\right)-2\sigma \left(C_{r}^{\prime \prime }\right)\leq 0\;or\;dist\left(C_{c}^{\prime },C_{c}^{\prime \prime }\right)-2\sigma \left(C_{c}^{\prime }\right)-2\sigma \left(C_{c}^{\prime \prime }\right)\leq 0$$

where *dist*(*w*, *z*) is the Euclidean distance between the centroids of the clusters *w *and *z *and *σ*(*w*) is the standard deviation of the cluster *w *(see Figure 5(b)). Since row and column objects are represented as vectors, the centroid of a cluster is computed in the classical way. The standard deviation for row and column objects is computed as $\sigma \left(C_{r}\right)=\sqrt{\frac{1}{\left(|C_{r}|-1\right)}\sum_{x\in C_{r}}euclidean\text{\_}distance\left(x,centroid\left(C_{r}\right)\right)}$ and $\sigma \left(C_{c}\right)=\sqrt{\frac{1}{\left(|C_{c}|-1\right)}\sum_{x\in C_{c}}euclidean\text{\_}distance\left(x,centroid\left(C_{c}\right)\right)}$ respectively.

Intuitively, conditions in (5) state that two biclusters are candidates for merging if they are close enough according to at least one dimension. By considering the factor 2 for *σ*(*w*), we include in each sphere about 95.4% of the objects of the corresponding cluster, as a consequence of Chebyshev's inequality.

If a pair of biclusters *C'*, *C" *is a candidate for merging, a further quality constraint should be satisfied on the bicluster *C''' *obtained by merging them. In particular, merging is performed as follows:

(6) $$C_{r}^{\prime \prime \prime }=C_{r}^{\prime }\cup C_{r}^{\prime \prime }\;C_{c}^{\prime \prime \prime }=C_{c}^{\prime }\cup C_{c}^{\prime \prime }$$

and the quality constraint that must be satisfied is *q*(*C'''*, *A*) >*α*, where *α *allows the user to decide the minimum cohesiveness value that each bicluster obtained after a merging step has to satisfy. Low values of α facilitate merging at the price of low cohesive biclusters.

**Figure 6:**
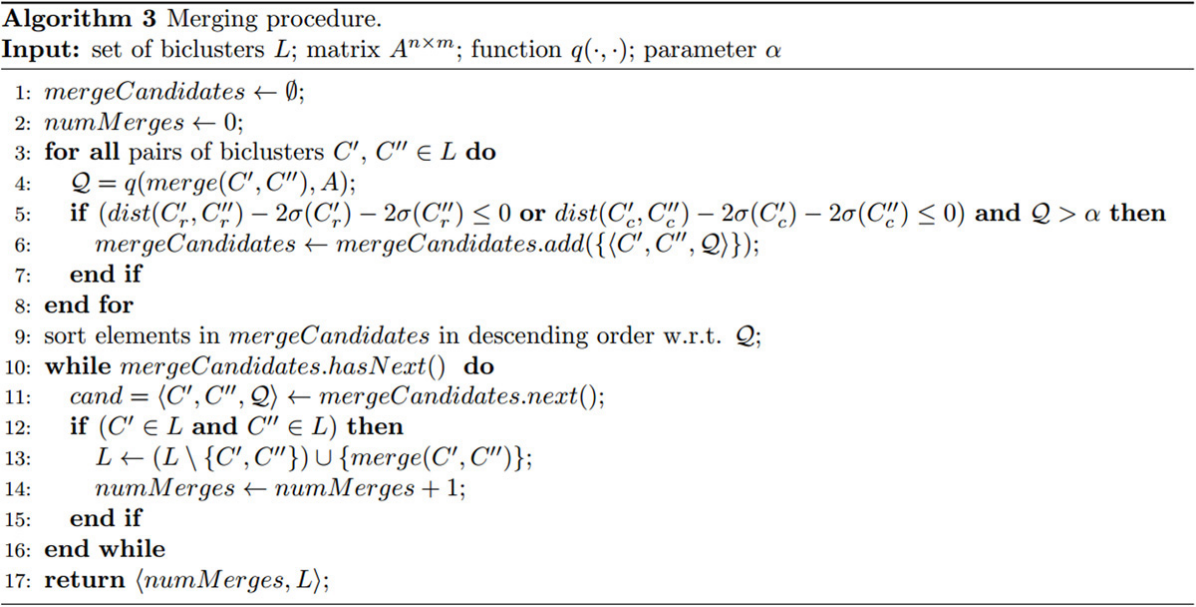
**Merging procedure**. High level description (in pseudocode) of the iterative merging phase of the proposed algorithm.

As in the overlap identification step, in order to obtain a result which is independent of the order in which pairs of biclusters are analyzed, merging is actually performed at the end of the procedure. Obviously, a bicluster could be a candidate for more than one merging. In this case, we actually perform the merging whose resulting bicluster has the maximum cohesiveness (see Figure 6).

It is noteworthy that the combination of our overlap identification and merging procedures allow us to consider both the density of biclusters and the distance among the objects, thus combining the major properties which classical clustering algorithms are based on.

### Ranking biclusters

Ranking of biclusters is based on the *p*-values of a statistical test which aims to evaluate the hypothesis that the mRNAs which belong to a specific bicluster are, on average, more functionally similar to other mRNAs in the same bicluster than to mRNAs which belong to other biclusters.

The functional similarity between two genes is evaluated by means of the *SimGIC *measure [33], which is a semantic similarity measure computed according to the genes' annotations in GO. As in [34], we consider the *similarity *as a special case of *relatedness *that is tied to the likeness (in the shape or form, e.g. on the basis of *is*-*a *relations) of concepts. *SimGIC *is proved to show high values of "resolution", that is, the relative intensity with which variations in the sequence similarity scale are translated into the semantic similarity scale. Moreover, as recognized in [33], in the case of GO (as in many other biological ontologies) Information Content-based approaches (like *SimGIC*), which estimate a term's specificity from its usage frequency within a given corpus, are more adequate than alternative approaches that typically infer a term's specificity from its depth in the graph. In fact, in the case of GO, the specificity is poorly related with the depth in the graph. For instance, the terms *binding *and *translation regulator activity *are at the same depth, but the latter is both semantically more complex and biologically more specific. *SimGIC *is defined according to the following formula:

(7) $$SimGIC\left(x_{1},x_{2}\right)=\frac{\sum_{t\in GO\left(x_{1}\right)\cap GO\left(x_{2}\right)}IC\left(t\right)}{\sum_{t\in GO\left(x_{1}\right)\cup GO\left(x_{2}\right)}IC\left(t\right)}$$

where *GO*(*x*) represents the set of GO terms which *x *is associated to and *IC*(*t*)= − log *p*(*t*) is the negative log-likelihood of the term *t *computed on the basis of the prior probability *p*(*t*) of *t*. *p*(*t*) is estimated as the percentage of genes associated to the term *t*, according to the UniProt Homo sapiens GO annotations. It is noteworthy that, although we used UniProt Homo sapiens GO annotations, in HOCCLUS2 other sets of annotations can be used.

**Figure 7:**
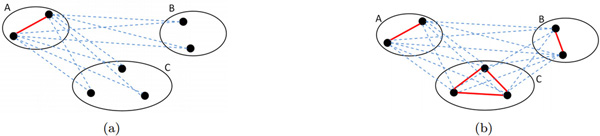
**Statistical tests for ranking and biclusters evaluation**. (a) **Ranking: **Average intra-bicluster similarity for bicluster *A *(on red edges) vs. average interbicluster similarity (on blue dashed edges). (b) **Biclustering Evaluation: **Average intra-bicluster similarity (on red edges) vs. average inter-bicluster similarity (on blue dashed edges).

The statistical test we consider is the classical one-tailed Student's *t *test that allows us to evaluate the null hypothesis *H*_0 _: *µ*_0_(*C*) = *µ*(*L*, *C*) against the alternative hypothesis *H*_1 _: *µ*_0_(*C*) >*µ*(*L*, *C*), where *µ*_0_(*C*) is the mean of the intra-bicluster functional similarities for the bicluster *C *and *µ*(*L*, *C*) is the mean of the inter-bicluster functional similarities between the bicluster *C *and the list *L *\{*C*}, i.e. the other biclusters belonging to the same hierarchy level of *C *(see Figure 7(a)). *µ*_0_(*C*) and *µ*(*L*, *C*) are defined as:

(8) $$\mu_{0}\left(C\right)=\frac{1}{|C_{r}|*\left(|C_{r}|-1\right)}\underset{x_{1}\in C_{r},x_{2}\in C_{r,}x_{1}\neq x_{2}}{\sum }SimGIC\left(x_{1},x_{2}\right)$$

(9) $$\mu \left(C,L\right)=\frac{1}{|L|-1}\underset{C^{\prime }\in L,C^{\prime }\neq C}{\sum }\left(\frac{\sum_{x_{1}\in \left(C_{r}\backslash {C^{\prime }}_{r}\right),x_{2}\in \left({C^{\prime }}_{r}\backslash C_{r}\right)}SimGIC\left(x_{1},x_{2}\right)}{\left|C_{r}\backslash {C^{\prime }}_{r}|*|{C^{\prime }}_{r}\backslash C_{r}\right|}\right)$$

This test is used to identify the *p*-value associated to the hypotheses to be verified. In particular, the lower the *p*-value, the lower the probability that *H*_0 _is rejected when *H*_0 _is true. Therefore, the lower the *p*-value, the higher the difference between the average intra-functional similarity and the average inter-functional similarity. These considerations make the *p*-value appropriate to be used in ranking biclusters in *L*, thus simplifying the identification of the most significant biclusters.

Since we compute SimGIC according to two different hierarchies of GO, that is, Molecular Function and Biological Process, we are able to obtain two different rankings of biclusters.

### Time complexity

The time complexity of the algorithm depends on the time complexity of each single phase.

As regards the initial biclustering, we first consider the miRNA to mRNA direction. The time complexity of *get*_*set*_*of*_*one*_*miRNA*_*bicliques*(*Vr*,*Vc*, *A*, β) is *O*(m * *n*) where *m *is the number of miRNAs and *n *is the number of mRNAs. At the first iteration, we have average time complexity:

$$O\left(\underset{\left(a\right)}{\underset{︸}{\frac{m*\left(m-1\right)}{2}}}\right)\left(\underset{\left(b\right)}{\underset{︸}{2+2avg\text{\_}mrna}}+\underset{\left(c\right)}{\underset{︸}{\left(2avg\text{\_}mrna\right)}}\right)+\left(\underset{\left(d\right)}{\underset{︸}{\frac{m*\left(m-1\right)}{2}*\text{log}\frac{m*\left(m-1\right)}{2}}}\right)=O\left(m^{2}*avg\text{\_}mrna\right)$$

where (a) is due to the pairwise comparison of one-miRNA bicliques, (b) is the cost of the union of miRNAs and intersection of mRNAs in two bicliques (we have 1 miRNA and *avg*_*mrna *mRNAs in one biclique), (c) is due to the computation of the cohesiveness function q and (d) is due to the identification of the best pair to be considered for aggregation (after sorting). Similarly, for the remaining iterations, we have:

$$\begin{array}{c} O\left[\underset{\left(a\right)}{\underset{︸}{m}}*\left(\underset{\left(b\right)}{\underset{︸}{m*\left(2avg\text{\_}mrna+2avg\text{\_}mrna+avg\text{\_}mrna*avg\text{\_}mrna\right)}}+\underset{\left(c\right)}{\underset{︸}{m*\text{log}(m*(m-1)/2}}\right)\right]=\\ \;\;O\left(m^{2}*\left(avg\text{\_}mirna*avg\text{\_}mrna\right)\right) \end{array}$$

where (a) represents the maximum number of iterations, (b) represents the cost of updating the candidate pairs of bicliques for aggregation at the light of the biclique added in the previous iteration and (c) represents the cost of adding the newly created candidates in *aggregationCandidates *(see Figure 3).

This analysis indicates that the cost of identifying the initial set of biclusters (by considering both directions) is *O*(2 * m * *n *+ (*m*^2 ^+ *n*^2^) * *avg*_mirna * *avg*_*mrna*), that is, since *n *≫ *m*:

(10) $$O\left(n^{2}*avg\text{\_}mirna*avg\text{\_}mrna\right)$$

In the overlap identification, at the first iteration (worst case), SMO is applied m * (*m *− 1)/2 times for each dimension (we assume that *m *is the number of biclusters after the initial biclustering). On average, the number of objects involved in each execution of SMO for row (column) objects is *avg*_*mirna *(*avg*_*mrna*). Therefore the cost of the overlap identification is: *O *(m * (*m *− 1) * (*avg*_mirna * *l*_1 _+ *avg*_ mrna * *l*_2_)) = *O *(*m*^2 ^* (*avg*_mirna * *l*_1 _+ *avg *_mrna * *l*_2_)) where *avg*_mirna*l_1_+*avg*_mrna*l_2 _is the cost of SMO and *l*_1 _and *l*_2 _are the number of candidate support vectors during the training phase (*l*_1 _≤ *avg*_*mirna*, *l*_2 _≤ *avg *_*mrna*).

The time complexity of the merging phase is: *O *(m * (*m - *1)/2 * *avg*_mirna * *avg*_*mrna*) = *O *(*m*^2 ^* *avg*_mirna * *avg*_*mrna*) where *avg*_mirna * *avg*_*mrna *is due to the cohesiveness function *q*.

Since the computation of the cohesiveness function *q *is exactly *avg*_mirna * *avg*_*mrna*, whereas the cost of the execution of SMO on both dimensions, in the worst case (*l*_1 _= *avg*_ *mirna *and *l*_2 _= *avg*_*mrna*), is *O*(*avg*_*mirna*^2 ^+ *avg*_*mrna*^2^), we approximate the complexity of a single iteration of the algorithm in Figure 2 to:

(11) $$O\left(m^{2}*\left(avg\text{\_}mirna^{2}+avg\text{\_}mrna^{2}\right)\right)$$

The time complexity of the ranking phase is:

(12) $$O\left(n*\left(n-1\right)*S/2+k*m\text{log}m\right)=O\left(S*n^{2}\right)$$

where n * (n − 1) * *S*/2 is the cost for computing all the possible *SimGIC *values (*S *is the cost for a single *SimGIC *value) and k * *m *log *m *is the cost of sorting all extracted biclusters for all the hierarchy levels (*k*).

By combining (10), (11) and (12), the time complexity of the complete algorithm is: *O *[*n*^2 ^* (*avg*_mirna * *avg*_*mrna*) + u * *m*^2 ^* (*avg*_*mirna*^2 ^+ *avg_mrna*^2^) + S * *n*^2^] where *u >*0 is the number of iterations of the algorithm in Figure 2. Since in the experiments we observed that the main cycle requires much more time than initial biclustering and ranking, it is reasonable to say that the actual time complexity is *O*(u * *m*^2 ^* (*avg*_*mirna*^2 ^+ *avg*_*mrna*^2^)). This complexity significantly depends on *avg*_*mirna *and *avg*_*mrna*, i.e. on the density of the matrix *A*. Moreover, due to the worst case assumptions, the analysis appears to be too pessimistic with respect to the actual times measured during the experiments.

## Results and discussion

In order to evaluate HOCCLUS2, we have considered as data sources a set of experimentally verified miRNA:mRNA interactions, i.e. miRTarBase [12], as well as the set of miRNAs target predictions in mirDIP [11]. These data sources have been used to obtain six distinct datasets (described later).

The main goal of this experimental evaluation is twofold: we empirically prove that the extracted biclusters preserve high values of cohesiveness and we evaluate extracted biclusters in order to empirically assess their biological significance. Moreover, we show the ability of HOCCLUS2 in ranking extracted biclusters.

Experiments have been performed with different values of *α *and β in order to evaluate their effect on the obtained biclusters. In the case of miRTarBase, we compare HOCCLUS2 with HOCCLUS (which uses METIS for the initial biclustering) [16] and ROCC [15]. In the case of mirDIP, we compare HOCCLUS2 with METIS [27] and ROCC. We cannot compare HOCCLUS2 with HOCCLUS on mirDIP because of the huge amount of non-linearly separable biclusters returned by METIS (with many similar miRNAs and mRNAs) that inhibit HOCCLUS from completing the mining process in reasonable running time. For fair comparison, METIS is asked to return the same number of biclusters returned by our initial biclustering algorithm.

### Datasets

miRTarBase (ver. 2.5) [12] contains 4,270 experimentally verified miRNA-target interactions between 669 miRNAs and 2,533 target genes among 14 species. In our study, we only consider the human dataset. From this dataset (hereafter *miRTarBase*), we have generated an additional dataset (*miRTarBase_filt_*), that contains only mRNAs which are annotated in GO, according to both Molecular Function (MF) and Biological Process (BP) hierarchies, and miRNAs that, once mRNAs have been filtered, are still connected to the remaining mRNAs. For both *miRTarBase *and *miRTarBase_filt_*, interaction scores are binary (yes = 1/no = 0). Table 1 provides additional details on *miRTarBase *and *miRTarBase_filt_*.

**Table 1:** Properties of considered datasets

Dataset	Data Source	No. interactions	No. miRNA	No. mRNA	GO Filter	Weighting
*miRTarBase *	miRTarBase	2,881	301	1,730		Binary
*miRTarBase_filt _*	miRTarBase	2,336	287	1,291	•	Binary
*mirDIP *	mirDIP	307,075	703	13,714		Average
*FmirDIP *	mirDIP	307,075	703	13,714		F-Score
*mirDIP_filt _*	mirDIP	207,011	703	8,495	•	Average
*FmirDIP_filt _*	mirDIP	207,011	703	8,495	•	F-Score

mirDIP [11] integrates twelve miRNA prediction datasets from seven miRNA prediction databases. In this study, we consider only predictions extracted using TargetScan Conserved, PITA Top Hits and picTar 5-way which, according to [11], provide a relatively low number of false positives (when compared to others) without affecting recall. We have not included additional prediction algorithms in order to minimize collinearity problems [35] in the combined predictions. Indeed, in order to obtain interaction scores, we (linearly) combine the standardized scores returned by single algorithms. In this combination, the consideration of the same features (e.g. conservation, site accessibility, free energy of duplex) multiple times may negatively affect the final score. This means that we have only considered the best prediction algorithms with the smallest overlap in the considered characteristics. From the original mirDIP we have generated 4 datasets: *mirDIP*, *FmirDIP*, *mirDIP_filt _*and *FmirDIP_filt_*. *mirDIP *contains all the predictions obtained by at least one of the considered prediction algorithms. In this case, interaction scores are obtained as the average of the standardized scores returned by each algorithm. *FmirDIP *is similar to *mirDIP*, except in the fact that the interaction scores are obtained according to a weighted average, where weights correspond to F-score values reported in [11] which represent a degree of "reliability" of the predictions of each algorithm. *mirDIP_filt _*and *FmirDIP_filt _*have been obtained from *mirDIP *and *FmirDIP *(respectively), by filtering out mRNAs whose genes are not included in GO. A summary of all considered datasets is reported in Table 1.

### Evaluation measures

Biclusters are evaluated on the basis of the average biclustering cohesiveness, which measures the average strength of the intra-biclusters connections: $\mu_{q}\left(L_{j},A\right)=\frac{1}{\sum_{C_{i}\in L_{j}}\left|C_{i}\right|}\sum_{C_{i}\in L_{j}}\left|C_{i}\right|q\left(C_{i},A\right)$, where *L_j _*is the set of biclusters obtained at the *j*-th hierarchy level. In addition to *µ_q_*(), we also use an evaluation measure which is based on statistical properties of the obtained biclusters. In particular, we use the independent two-sample Student's *t *test to evaluate the null hypothesis $H_{0}:\mu_{0}^{\prime }\left(L_{j}\right)=\mu^{\prime }\left(L_{j}\right)$ against the alternative hypothesis $H_{1}:\mu_{0}^{\prime }\left(L_{j}\right)=\mu^{\prime }\left(L_{j}\right)$, where $\mu_{0}^{\prime }\left(L_{j}\right)$ is the average intra-bicluster functional similarity $\mu_{0}^{\prime }\left(L_{j}\right)=\frac{1}{\left|L_{j}\right|}\sum_{C\in L_{j}}\mu_{0}\left(C\right)$ and $\mu^{\prime }\left(L_{j}\right)$ is the average inter-bicluster functional similarity defined as follows:

(13) $$\mu^{\prime }\left(L_{j}\right)=\frac{1}{|L_{j}|*\left(|L_{j}|-1\right)}\underset{C^{\prime }\in L_{j},C^{\prime \prime }\in L_{j},C^{\prime }\neq C^{\prime \prime }}{\sum }\left(\frac{\sum_{x_{1}\in \left({C^{\prime }}_{r}\backslash {C^{\prime \prime }}_{r}\right),x_{2}\in \left({C^{\prime \prime }}_{r}\backslash {C^{\prime }}_{r}\right)}SimGIC\left(x_{1},x_{2}\right)}{\left|{C^{\prime }}_{r}\backslash {C^{\prime \prime }}_{r}|*|{C^{\prime \prime }}_{r}\backslash {C^{\prime }}_{r}\right|}\right)$$

The lower the *p*-value, the higher the difference between the average intra-functional similarity and the average inter-functional similarity (see Figure 7(b)). As in the ranking phase, we use both GO Biological Process (BP) and GO Molecular Function (MF) hierarchies to compute *SimGIC*. Henceforth we will refer to the *p*-values computed on BP and MF as *p_BP _*and *p_MF _*, respectively.

In the following, we report results for the first level of the hierarchy (i.e. the result of the initial biclustering), for the last level of the hierarchy (i.e. *L_k_*) and for the "best level" of the hierarchy. The best level is the one for which we have the minimum value of $\frac{p_{BP}+p_{MF}}{2}$. When more than one level has the same $\frac{p_{BP}+p_{MF}}{2}$ value, the one with the highest *µ_q _*is considered. Experiments are run on a 4 Intel CPUs @4Ghz system.

### miRTarBase

Tables 2 and 3 report results obtained with HOCCLUS2, HOCCLUS and ROCC on miRTarBase datasets. From these tables, it is possible to see that at the highest levels of the hierarchy, results significantly depend on the values of the parameter *α*. As expected, the higher the *α *value, the higher the *µ_q _*value. An important result comes from the very low values of *p_MF _*and *p_BP_*. This statistically confirms (at a confidence level of 99%) that mRNAs which belong to the same biclusters are, on average, more functionally similar than mRNAs which belong to different biclusters. Moreover, *p_MF _*and *p_BP _*results are monotonic with respect to *µ_q _*(and *α*). This confirms that *µ_q _*is a valid measure to evaluate the quality of extracted biclusters. Another result is that at higher levels of the hierarchy, HOCCLUS2 is generally able to significantly reduce (especially for high values of *α*) the number of biclusters without a significant loss in the *p_MF_*, *p_BP _*and *µ_q_*.

**Table 2:** miRTarBase results

*α*	N(%/%)	level 1					max level						best level					time (*s*)
			
		#cc	*p_MF_*	*p_BP_*	*μ_q_*		lev	#cc	*p_MF_*	*p_BP_*	*μ_q_*		lev	#cc	*p_MF_*	*p_BP_*	*μ_q_*	
**HOCCLUS2**																		

0.1	10/37	75	1.000	1.000	1.00		6	5	0.281	0.289	0.13		2	38	0.000	0.000	0.72	10
						
0.2							5	10	0.064	0.060	0.24		2	38	0.000	0.000	0.72	9
						
0.3							4	18	0.000	0.000	0.40		2	38	0.000	0.000	0.72	9
						
0.4							4	23	0.000	0.000	0.49		2	38	0.000	0.000	0.72	10
						
0.5							3	33	0.000	0.000	0.66		2	38	0.000	0.000	0.72	9

**HOCCLUS (with METIS)**																		

0.1	100/100	75	0.000	0.003	0.19		3	51	0.013	0.031	0.09		1	75	0.000	0.003	0.19	51
						
0.2							2	73	0.000	0.007	0.14		1	75	0.000	0.003	0.19	82
						
0.3							1	75	0.000	0.003	0.19		1	75	0.000	0.003	0.19	94
						
0.4							1	75	0.000	0.003	0.19		1	75	0.000	0.003	0.19	94
						
0.5							1	75	0.000	0.003	0.19		1	75	0.000	0.003	0.19	92

**ROCC**																		

-	35/17	198	0.231	1.000	0.01		-	-	-	-	-		-	-	-	-	-	149

*miRTarBase *results. N(%/%) represents the percentage of biclustered mRNAs and miRNAs. #cc is the number of biclusters. *lev *represents the level number. Results do not vary at the first level since it does not depend on the *α *value. β does not influence results because of binary interaction scores.

**Table 3:** miRTarBase_fil__t _results

*α*	N(%/%)	level 1					max level						best level					time (*s*)
			
		#cc	*p_MF_*	*p_BP_*	*μ_q_*		lev	#cc	*p_MF_*	*p_BP_*	*μ_q_*		lev	#cc	*p_MF_*	*p_BP_*	*μ_q_*	
**HOCCLUS2**																		

0.1	12/36	72	0.000	0.000	1.00		6	5	0.099	0.125	0.14		1	72	0.000	0.000	1.00	9
						
0.2							5	10	0.011	0.015	0.27		1	72	0.000	0.000	1.00	8
						
0.3							4	17	0.009	0.002	0.41		1	72	0.000	0.000	1.00	8
						
0.4							4	24	0.000	0.002	0.55		1	72	0.000	0.000	1.00	8
						
0.5							3	28	0.000	0.001	0.61		1	72	0.000	0.000	1.00	8

**HOCCLUS (with METIS)**																		

0.1	100/100	72	0.000	0.015	0.21		3	43	0.008	0.043	0.10		2	47	0.003	0.008	0.11	30
						
0.2							2	68	0.000	0.006	0.14		2	68	0.000	0.006	0.14	54
						
0.3							1	72	0.000	0.015	0.21		1	72	0.000	0.015	0.21	70
						
0.4							1	72	0.000	0.015	0.21		1	72	0.000	0.015	0.21	71
						
0.5							1	72	0.000	0.015	0.21		1	72	0.000	0.015	0.21	71

**ROCC**																		

-	41/16	198	1.000	1.000	0.01		-	-	-	-	-		-	-	-	-	-	89

*miRTarBase*_filt _results. N(%/%)represents the percentage of biclustered mRNAs and miRNAs. #cc is the number of biclusters. *lev *represents the level number. Results do not vary at the first level since it does not depend on the *α *value. β does not influence results because of binary interaction scores.

An additional important result is the number of biclustered miRNAs and mRNAs (N), which gives an indication of the number of isolated objects in miRTarBase that are considered noise by HOCCLUS2. Finally, by comparing results in the two tables, it is possible to say that considering only filtered data improves both the significance of the statistical analysis (*p_MF _*and *p_BP _*) and the cohesiveness of biclusters.

Regarding comparison with other systems, HOCCLUS2 performs significantly better than ROCC, which, as previously stated, is not designed to work with miRNA:mRNA interactions. Moreover, HOCCLUS2 always outperforms HOCCLUS in terms of cohesiveness and, at the best hierarchy level, in terms of *p_MF _*and *p_BP_*. In the last hierarchy level (max level), better performances of HOCCLUS with *α *= 0.1 and *α *= 0.2 can be motivated by the huge number of extracted biclusters (e.g. 52 vs. 5 extracted by HOCCLUS2, with *α *= 0.1). As regards the number of noise objects, HOCCLUS2 has biclustered less mRNAs and more miRNAs than ROCC. In this respect, the behavior of HOCCLUS2 is preferable, since the number of miRNAs is generally significantly lower than the number of mRNAs (HOCCLUS2 better deals with the relational imbalance). Moreover, while the possibility to discard noise objects helped HOCCLUS2 to achieve a very high value of cohesiveness, ROCC obtained poor results (*µ_q _*= 0.01).

Running times show that HOCCLUS2 outperforms both HOCCLUS and ROCC. This confirms that the reported complexity analysis is too pessimistic and that HOCCLUS2 extracts biclusters in a reasonable time.

### mirDIP

Tables 4, 5, 6 and 7 report results obtained on the mirDIP datasets. All the considerations about the monotonicity between *α *and *µ_q _*and the capability of HOCCLUS2 to extract cohesiveness-preserving hierarchies reported for *miRTarBase *datasets are valid also for the *mirDIP *datasets. However, in this case, *p_MF _*and *p_BP _*are monotonic with respect to the cohesiveness only in the case of filtered datasets. This can be explained by the high number of missing GO annotations (for about 40% of biclustered mRNAs) in *mirDIP *and *FmirDIP *datasets which makes *p_MF _*and *p_BP _*not completely reliable indicators of the biclusters' quality. In these cases, i.e. when the algorithm cannot calculate reliable values of *p_MF _*and *p_BP_*, *µ_q _*should be considered the primary indicator for the evaluation.

**Table 4:** mirDIP results

*α*	*β*	N(%/%)	level 1					max level						best level					time (*s*)
				
			#cc	*p_MF_*	*p_BP_*	*μ_q_*		lev	#cc	*p_MF_*	*p_BP_*	*μ_q_*		lev	#cc	*p_MF_*	*p_BP_*	*μ_q_*	
**HOCCLUS2**																			

0.1	0.3	43/87	700	1.000	1.000	0.49		9	56	0.000	0.000	0.12		2	350	0.000	0.000	0.41	5797
							
0.2								7	183	0.000	0.000	0.24		3	210	0.000	0.000	0.31	6416
							
0.3								5	355	1.000	1.000	0.36		1	700	1.000	1.000	0.49	7783

0.1	0.4	36/86	619	1.000	1.000	0.52		8	41	0.411	0.331	0.11		3	155	0.004	0.009	0.32	4089
							
0.2								7	144	0.006	0.001	0.24		7	144	0.006	0.001	0.24	4816
							
0.3								6	274	1.000	1.000	0.35		1	619	1.000	1.000	0.52	6123

0.1	0.5	25/81	599	1.000	1.000	0.58		8	34	0.284	0.273	0.12		4	77	0.345	0.167	0.27	3439
							
0.2								7	101	0.315	0.146	0.23		5	108	0.257	0.112	0.26	3958
							
0.3								6	202	1.000	0.221	0.34		5	205	1.000	0.206	0.35	4361

**METIS**																			

-	-	100/100	700	1.000	1.000	0.36		-	-	-	-	-		-	-	-	-	-	50
							
-	-		619	1.000	1.000	0.40		-	-	-	-	-		-	-	-	-	-	51
							
-	-		599	1.000	1.000	0.35		-	-	-	-	-		-	-	-	-	-	52

**ROCC**																			

-	-	1/1	122	1.000	1.000	0.01		-	-	-	-	-		-	-	-	-	-	16531

*mirDIP *results. N(%/%)represents the percentage of biclustered mRNAs and miRNAs. #cc is the number of biclusters. *lev *represents the level number.

**Table 5:** FmirDIP results

*α*	*β*	N(%/%)	level 1				max level					best level					time (*s*)
				
			#cc	*p_MF_*	*p_BP_*	*μ_q_*	lev	#cc	*p_MF_*	*p_BP_*	*μ_q_*	lev	#cc	*p_MF_*	*p_BP_*	*μ_q_*	
**HOCCLUS2**																	

0.1	0.3	45/88	758	1.000	1.000	0.50	9	57	0.023	0.005	0.11	2	379	0.000	0.000	0.41	7319
							
0.2							7	194	0.016	0.004	0.25	3	221	0.001	0.000	0.31	9022
							
0.3							6	374	1.000	1.000	0.36	1	758	1.000	1.000	0.50	9940

0.1	0.4	37/85	667	1.000	1.000	0.54	7	42	0.434	0.206	0.11	4	58	0.094	0.016	0.21	4760
							
0.2							6	145	0.096	0.004	0.24	5	148	0.053	0.004	0.25	5415
							
0.3							5	273	1.000	1.000	0.34	1	667	1.000	1.000	0.54	6567

0.1	0.5	27/81	622	1.000	1.000	0.60	8	35	0.311	0.346	0.12	3	156	0.151	0.263	0.37	3792
							
0.2							7	105	0.221	1.000	0.24	3	168	0.123	0.298	0.38	4121
							
0.3							6	205	0.374	1.000	0.36	2	314	0.256	1.000	0.50	4685

**METIS**																	

-	-	100/100	758	1.000	1.000	0.29	-	-	-	-	-	-	-	-	-	-	50
		
-	-		667	1.000	1.000	0.39	-	-	-	-	-	-	-	-	-	-	51
		
-	-		622	1.000	1.000	0.35	-	-	-	-	-	-	-	-	-	-	50

**ROCC**																	

-	-	1/1	122	1.000	1.000	0.01	-	-	-	-	-	-	-	-	-	-	16764

*FmirDIP *results. N(%/%)represents the percentage of biclustered mRNAs and miRNAs. #cc is the number of biclusters. *Lev *represents the level number.

**Table 6:** mirDIP_filt _results

*α*	*β*	N(%/%)	level 1				max level					best level					time (*s*)
				
			#cc	*p_MF_*	*p_BP_*	*μ_q_*	lev	#cc	*p_MF_*	*p_BP_*	*μ_q_*	lev	#cc	*p_MF_*	*p_BP_*	*μ_q_*	
**HOCCLUS2**																	

0.1	0.3	46/86	528	0.000	0.000	0.49	8	40	0.027	0.127	0.12	1	528	0.000	0.000	0.49	2031
							
0.2							6	135	0.001	0.000	0.25	1	528	0.000	0.000	0.49	2225
							
0.3							5	269	0.000	0.000	0.36	1	528	0.000	0.000	0.49	2824

0.1	0.4	40/85	500	0.000	0.000	0.52	7	35	0.056	0.045	0.12	1	500	0.000	0.000	0.52	1627
							
0.2							6	121	0.000	0.000	0.25	1	500	0.000	0.000	0.52	1870
							
0.3							5	225	0.000	0.000	0.35	1	500	0.000	0.000	0.52	2213

0.1	0.5	29/79	503	1.000	1.000	0.58	8	30	0.061	0.215	0.12	8	30	0.061	0.215	0.12	1495
							
0.2							6	92	0.485	1.000	0.24	2	252	0.188	0.110	0.49	1582
							
0.3							6	178	0.095	0.215	0.36	4	184	0.046	0.165	0.38	1948

**METIS**																	

-	-	100/100	528	1.000	1.000	0.34	-	-	-	-	-	-	-	-	-	-	26
		
-	-		500	1.000	1.000	0.30	-	-	-	-	-	-	-	-	-	-	27
		
-	-		503	1.000	1.000	0.31	-	-	-	-	-	-	-	-	-	-	24

**ROCC**																	

-	-	-	-	-	-	-	-	-	-	-	-	-	-	-	-	-	-

*mirDIP_filt _*results. N(%/%)represents the percentage of biclustered mRNAs and miRNAs. #cc is the number of biclusters. *lev *represents the level number. ROCC results are not reported for filtered datasets since it raised an error during the execution and the biclustering process could not terminate.

**Table 7:** FmirDIP_filt _results

*α*	*Β*	N(%/%)	level 1				max level					best level					time (*s*)
				
			#cc	*p_MF_*	*p_BP_*	*μ_q_*	lev	#cc	*p_MF_*	*p_BP_*	*μ_q_*	lev	#cc	*p_MF_*	*p_BP_*	*μ_q_*	
**HOCCLUS2**																	

0.1	0.3	50/86	561	0.000	0.000	0.50	8	39	0.001	0.048	0.12	1	561	0.000	0.000	0.50	2287
							
0.2							7	138	0.019	0.000	0.24	2	282	0.012	0.000	0.40	2666
							
0.3							5	266	0.000	0.000	0.36	1	561	0.000	0.000	0.50	3423

0.1	0.4	42/84	522	0.000	0.000	0.54	7	35	0.074	0.117	0.12	1	522	0.000	0.000	0.54	1845
							
0.2							6	114	0.006	0.002	0.24	1	522	0.000	0.000	0.54	2025
							
0.3							5	217	0.000	0.000	0.35	1	522	0.000	0.000	0.54	2373

0.1	0.5	31/79	513	0.028	1.414	0.60	7	29	0.370	0.310	0.12	2	258	0.002	0.066	0.49	1597
							
0.2							6	90	0.137	0.180	0.24	2	258	0.002	0.066	0.49	1706
							
0.3							6	172	0.001	1.000	0.35	2	260	0.002	0.079	0.49	2052

**METIS**																	

-	-	100/100	465	1.000	1.000	0.35	-	-	-	-	-	-	-	-	-	-	27
		
-	-		522	1.000	1.000	0.35	-	-	-	-	-	-	-	-	-	-	25
		
-	-		515	1.000	1.000	0.34	-	-	-	-	-	-	-	-	-	-	26

**ROCC**																	

-	-	-	-	-	-	-	-	-	-	-	-	-	-	-	-	-	-

*FmirDIP_filt _*results. N(%/%)represents the percentage of biclustered mRNAs and miRNAs. #cc is the number of biclusters. *lev *represents the level number. ROCC results are not reported for filtered datasets since it raised an error during the execution and the biclustering process could not terminate.

By observing the differences between *mirDIP *and *FmirDIP *(or between *mirDIP_filt _*and *FmirDIP_filt_*) it is possible to say that, coherently with results reported in [11], HOCCLUS2 benefits from the F-score-based weighting of the interactions. Furthermore, when compared with other algorithms, HOCCLUS2 performs significantly better, in terms of cohesiveness, than ROCC and METIS. Additionally, ROCC and METIS are not able to extract significant biclusters in terms of *p_BP _*and *p_MF_*, whereas HOCCLUS2 is almost always able to extract actual functional biclusters for at least one level of the hierarchy.

As regards the number of noise objects, while ROCC has biclustered a very low number of miRNAs and mRNAs (9 and 101 respectively in *mirDIP*), obtaining a poor value of choesiveness (0.01), HOCCLUS2 has biclustered a reasonable number of objects for every considered values of β.

Running times show that HOCCLUS2 is always faster than ROCC. Moreover, although METIS requires significantly lower time, a detailed analysis reveals that the time for completing our initial biclustering step is comparable to that of METIS (we remind that METIS returns non-hierarchically organized biclusters). Similar to miRTarBase, also these results confirm that the reported worst case analysis is too pessimistic. Here, in addition, we demonstrate that HOCCLUS2 scales well also for huge datasets.

### Biological evaluation of extracted biclusters

In order to evaluate the ability of HOCCLUS2 to detect meaningful miRNAs:mRNAs functional relationships, we have first analyzed the results obtained from miRTarBase datasets and then compared them with the results obtained from mirDIP. In this analysis, we have selected biclusters to be analyzed only according to the ranking values returned by the algorithm. In this paper we focus the analysis on some of the biclusters which group miRNAs belonging to the miR-17-92 gene cluster, also known as oncomir-1 [36], and to its paralogs, miR-106b-25 and miR-106a-363. Table 8 reports the complete list of these biclusters, together with their properties. Moreover, some other examples of biclusters are provided to elucidate the usefulness of HOCCLUS2 in supporting biologists in the detection of multiple miRNAs functional interactions and in the identification of new potential targets. Functional analysis has been carried out by considering: *i*) structural and functional properties of miRNAs; *ii*) pathways mapping and statistical significance of gene enrichment in pathways; *iii*) the biological context of target genes. The main resource used for mapping gene in pathways is Reactome [37]. GeneCards [38] has been used for retrieving gene function information. Studies reported in the literature have been considered *i*) for retrieving information on miRNAs and validated miRNA:mRNA interactions and *ii*) for the discussion of the results.

**Table 8:** Biclusters containing the miR-17-92 gene cluster family

ID	miRNAs	mRNAs	miRNA GC	*p_MF_*	*p_BP_*	*q*
**Hierarchy level 2**						

6	mir-17, mir-20a	APP, BCL2, BMPR2, CCND1, CCND2, CDKN1A, E2F1, E2F3, MAP3K12, MAPK9, MEF2D, MYC, PTEN, RB1, RBL1, RBL2, RUNX1, SMAD4, TGFBR2, THBS1, VEGFA, WEE1	miR-17-92	3.96 E-6	3.37 E-7	1.0

22	mir-93, mir-106b	CDKN1A, E2F1, KAT2B, MAPK9, VEGFA	miR-106b-25	1.00	1.30 E-6	1.0

31	mir-20b, mir-106a	ARID4B, CDKN1A, HIPK3, MYLIP, VEGFA	miR-106a-363	1.00	1.00	1.0

58	mir-17, mir-21, mir-18a, mir-19b	NCOA3, PTEN	miR-17-92	1.00	1.00	1.0

66	mir-17, mir-19a, mir-20a, mir-92a	BMPR2, SMAD4, TGFBR2	miR-17-92	5.53 E-41	3.23 E-31	1.0

67	mir-19b, mir-20b, mir-92a, mir-106a	ARID4B, HIPK3, MYLIP	miR-17-92, miR106a-363	1.00	0.03	1.0

70	mir-17, mir-20a, mir-106a	CDKN1A, E2F1, RB1, RUNX1, VEGFA	miR-17-92, miR106a-363	5.77 E-4	1.99 E-9	1.0

72	mir-17, mir-20a, mir-106b	APP, CCND2, E2F3, MAPK9, RBL1, RBL2, WEE1	miR-17-92, miR-106b-25	1.00	5.00 E-24	1.0

**Hierarchy level 2**						

6-72	mir-17, mir-20a, mir-106b	APP, BCL2, BMPR2, CCND1, CCND2, CDKN1A, E2F1, E2F3, MAP3K12, MAPK9, MEF2D, MYC, PTEN, RB1, RBL1, RBL2, RUNX1, SMAD4, TGFBR2, THBS1, VEGFA, WEE1	miR-17-92, miR106b-25	1.90 E-5	6.46 E-5	0.8

22-70	mir-17, mir-93, mir-20a, mir-106a, mir-106b	CDKN1A, E2F1, KAT2B, MAPK9, RB1, RUNX1, VEGFA	miR-17-92, miR-106b-25, miR106a-363	1.60 E-5	1.07 E-7	0.8

58-66	mir-17, mir-21, mir-18a, mir-19a, mir-19b, mir-20a, mir-92a	BMPR2, NCOA3, PTEN, SMAD4, TGFBR2	miR-17-92	3.18 E-9	1.30 E-8	0.8

**Hierarchy level 3**						

6-72-22-70	mir-17, mir-93, mir-20a, mir-106a, mir-106b	APP, BCL2, BMPR2, CCND1, CCND2, CDKN1A, E2F1, E2F3, KAT2B, MAP3K12, MAPK9, MEF2D, MYC, PTEN, RB1, RBL1, RBL2, RUNX1, SMAD4, TGFBR2, THBS1, VEGFA, WEE1	miR-17-92, miR-106b-25, miR106a-363	2.46 E-4	1.56 E-4	0.6

Biclusters containing the miR-17-92 gene cluster family, extracted from *miRTarBase_filt _*with *α *= 0.2. The column *miRNA GC *contains the corresponding gene cluster in the miR-17-92 family.

#### Structural and functional properties of miR-17-92, miR-106b-25 and miR-106a-363

miR-17-92, miR-106b-25 and miR-106a-363 belong to a family of highly conserved miRNA gene clusters and have potent effects on many type of human cancers [39]. They are located on chromosome 13, 7 and X, respectively, and derive from duplications and mutations of a unique gene and from the loss of some miRNAs occurred during the early evolution of vertebrates. Each cluster is transcribed as polycistronic primary transcript that ultimately yields six mature miRNAs in miR-17-92 and miR-106a-363 (miR-17, miR-18a, miR-19a, miR-20a, miR-19b-1, miR-92a-1 in miR-17-92; miR-106a, miR-18b, miR-20b, miR-19b-2, miR-92a-2 and miR-363 in miR-106a-363) and three miRNAs in miR-106b-25 (miR-106-b, miR-93, miR-25). The high degree of conservation across different species suggests an important role of this miRNAs cluster family for coordinated regulation and function in many pathways and cellular processes.

The miR-17-92 gene cluster acts pleiotropically during both normal development and cancer progression. Depending on both cell type and physiological context, miR-17-92 can promote proliferation, increase angiogenesis, and sustain cell survival through the post-transcriptional repression of a number of target mR-NAs [39]. Different types of experimental evidences suggest the intriguing hypothesis that miRNAs in the miR-17-92 cluster may perform specific functions, either individually or in combination, in a coordinated rather than in an additive manner. A key feature of miR-17-92 is its property of being a potent inhibitor of the transforming growth factor-β (TGF-β) signaling. Ligands of the TGF-β superfamily are essential for the development and the adult tissue homeostasis, and the inactivation of TGF-β tumor suppression pathway is a main step in the development of a variety of human tumors [40]. Indeed, the miR-17-92 cluster is often activated in cancer cells and overexpression studies in gastrointestinal and other tumors reveal that both miR-17-92 and miR-106b-25 are able to inactivate the TGF-β tumor suppression pathway by interfering with the cell cycle arrest and apoptosis [40]. Although recent studies have greatly contributed to the elucidation of the miR-17-92 gene cluster family function and mechanism, the identity of all its targets remains still elusive and much work is still necessary to clarify miRNAs cooperative effects on signaling pathways. Moreover, the role of miR-106a-363 remains still obscure.

#### Validation of the functional consistency of extracted biclusters

The large amount of literature available on the miR-17-92 gene cluster family constitutes a reliable resource to verify the ability of our algorithm to discover actual biological functional interactions among miRNAs and their target genes belonging to the same bicluster. The rationale is that, if the results obtained on experimentally verified datasets are confirmed, there exists a real possibility that our biclustering algorithm is effective and that it could also work well on large datasets produced by prediction algorithms. This would allow us to uncover new potential gene functions and targeting interactions.

The aim of the analysis reported in this section is not to give a complete and exhaustive picture of all the possible discovered interaction networks, that would be impossible to report and that does not fit the aim of the present paper, but only to demonstrate that the system shows to be effective.

We have identified and analyzed a series of highly-ranked biclusters containing the miR-17-92 cluster family. Table 8 reports the list of miRNAs and relevant target genes for each of these biclusters. Biclusters at level number 1 are biclusters where all included genes are targeted by all the miRNAs grouped in the bicluster. At higher levels of the hierarchy, other miRNAs and targets are included at different values of cohesiveness suggesting miRNAs alternative multiple interactions. The identification of specific and yet overlapping functions of each component of the miR-17-92 cluster, can be obtained by comparing targets in each bicluster with those belonging to other related biclusters.

**Figure 8:**
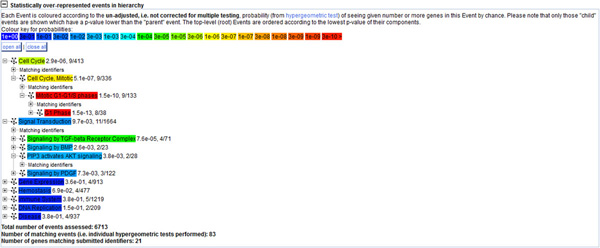
**Pathway mapping of the bicluster 6-72-22-70 in Reactome**. The figure shows the pathway mapping of the bicluster 6-72-22-70 in Reactome. Results in a tab-delimited format as provided by Reactome are reported in the Additional file 1. The functional mapping of the biclusters 6 and 6-72 is very similar to that of the bicluster 6-72-22-70. The difference is in the fact that KAT2B is only present in the bicluster 6-72-22-70.

Reactome-based mapping of biclusters 6 (hierarchy level 1), 6-72 (hierarchy level 2) and 6-72-22-70 (hierarchy level 3)(Figure 8), well matches the known functions of miR-17-92. Indeed, the most overrepresented events are cell cycle and signal transduction. In particular, as for cell cycle, the mitotic transition from the G1 to the S phase is represented with the lowest p-value (1.5 E-13) with 9 (E2F1, E2F3, RB1, CDKN1A, RBL2, CCND2, WEE1, RBL1, CCND1) out of 23 of the target genes involved in this pathway. Signal transduction pathway, with 11 (THBS1, PTEN, TGFBR2, VEGFA, CDKN1A, RBL1, KAT2B, APP, BMPR2, SMAD4, MYC) out of 23 genes involved, is represented with the lowest p-value (2.5 E-10) in the TGF-β signaling pathway including TGFBR2, SMAD4, MYC and RBL1. Other related signaling pathways with significant p-values are signaling by BMP (bone morphogenetic protein), AKT (Protein Kinase B), PDGF (platelet-derived growth factor); signaling by EGFR (epidermal growth factor receptor) in cancer and FGFR (fibroblast growth factor receptors) in disease. EGFR and FGFR signaling pathways depend on PTEN PIP3 activation in the AKT signaling, promoting cell survival and opposing apoptosis by a variety of routes. Gene expression and DNA replication pathways are also over-represented because influenced by two main downstream effectors of the TGF-β antiproliferative signaling pathway, SMAD4 and CDKN1A [40], and by RB1. SMAD4 is the common signaling transducer of TGF-β at nuclear level, while CDKN1A and RB1 are master regulators of cell cycle progression (negatively regulate the mitotic G1-S checkpoint). The analysis of biclusters that contribute to the bicluster 6-72-22-70 clarifies the individual contribution of miRNAs and target genes in the general picture above. The main difference between 6 (hierarchy level 1), 6-72 (hierarchy level 2) and 6-72-22-70 (hierarchy level 3) is the miRNA component. Biclustering at level 1 (i.e., bicluster 6) indicates that all the genes in the bicluster are unique targets of miR-17 and miR-20a, suggesting that only these two genes have a universal role whereas the others may have a pathway-specific activity. This observation contributes to clarify the general model that, in the attempt to explain the pleiotropic effect of miR-17-92, proposes that the entire gene cluster gives rise to a moderate down-regulation of a large number of mRNAs in each cell type, which collectively mediates its biological functions [39].

As for the targets component of these biclusters, KAT2B is the unique gene that is only present in bicluster 6-72-22-70 but not in bicluster 6 and 6-72. Looking for other biclusters containing KAT2B at level 1 of the hierarchy (Table 8), it is possible to see that it is present in the bicluster 22 and is cotargeted by miR-93 and miR-106b. KAT2B has not been included in the biclusters 6 because, differently from the other genes, it is not target of miR-17 and miR-20a. This is confirmed by a study on multiple myeloma pathogenesis [41] which demonstrates that, among over expressed miRNAs, miR-106b-25, but not miR-17-92, is able (through KAT2B) to indirectly control the tumor suppressor protein p53 in the multiple myeloma. Indeed, KAT2B is a histone acetyltransferase involved in the reversible acetylation of various transcriptional regulators, including the tumor suppressor protein p53. Activation of p53 mediated by KAT2B activates CDKN1A (a direct target and master effector of p53) that in turn induces the arrest of the cell cycle at the G1/S transition, and a series of other p53-dependent events such as DNA repair and apoptosis. Futhermore, this specific function of KAT2B would be mediated by the coordinate co-targeting of miR-181a, miR-181b and miR-32 [41]. The co-targeting of these last miRNAs on KAT2B is not included in biclusters 22, 22-70 and 6-72-22-70 but is included in biclusters 41 and 65 at level 1 and in bicluster 16-65 at level 2. These biclusters, although not statistically supported by GO, help to disclose new interaction networks. Indeed, in these biclusters (see Table 9), other important regulators of key steps of the cell cycle, TGF-β signaling pathway, cell growth, differentiation and apoptosis, are associated with KAT2B and with the co-targeting of miR-25, miR-32, miR-19a, miR-19b, miR181a and miR181b. Moreover, these biclusters, as they also contain BCL2, PTEN, BMPR2 and TGFBR2 (also present in bicluster 6-72-22-70), suggest that complex interaction networks involving miR-25, miR-32, miR-181a and miR-181b, may account for the diverse and multiple role of miR-17-92 gene cluster in the maintenance of cell homeostasis. In particular, in bicluster 65, KAT2B is associated, under the direct control of miR-25, miR-32 and miR-19a, with BCL2L11(BIM), the master downstream effector of TGF-β-depend apoptosis, and with PRMT5, a protein arginine methyltransferase that negatively regulates cell proliferation by epigenetic control of the RB family of tumor suppressor genes (RB1, RBL1 and RBL2), and that it is regulated by miR-19a, miR-25, miR-32, miR-92b and miR-96 [42]. The RB family members are known to regulate the expression of genes involved in G1/S transition through their interaction with the E2F transcription factors [43]. However, while transcription of RB1 is repressed in a cell cycle-dependent manner, the PRMT5-mediated inhibition of RBL1 and RBL2 appears to be associated, in leukemia and lymphoma cells transformation, with the deregulation of specific miRNAs [42]. RB1, RBL1 and RBL2 are all present in biclusters 6, 6-72 and 6-72-22-70 and, as shown in bicluster 6, they are all direct targets of miR-17 and miR-20a. However, as shown in biclusters 70 and 72, RB1 is co-targeted by miR-106a, whereas RBL1 and RBL2 are co-targeted by miR-106b. This suggests for miR-106a and miR-106b a functional specificity that could be responsible for the context-dependent response of RBs and of the other genes in these biclusters. Indeed, also E2F1 and E2F3, which are functionally related to RB1 and RBL1/RBL2 [44] (respectively), are coherently biclustered in biclusters 70 and 72. This indicates that functional relationships between E2Fs and RBs [44], as well as the different responses of the RB components (see [42]), may be due to a complex network of transcriptional machineries and regulatory negative feedbacks [39]. This complex network involves transcriptional factors (e.g., E2F1, E2F3, p53, VEGFA) regulating, and in turn regulated by, different components of the miR-17-92 cluster family in a cell type and context-dependent manner.

**Table 9:** Biclusters containing co-targeting on KAT2B

ID	*p_MF_*	*p_BP_*	q	miRNAs	mRNAs	mRNA Function	Reactome Mapping (p-value)
Hierarchy level 1							

41	0.40	0.05	1.00	miR-181a, miR-181b	NLK	Serine/threonine-protein kinase that regulates a number of transcription factors with key roles in cell fate determination. Positive effector of the non-canonical Wnt signaling pathway and negative regulator of the canonical Wnt/beta-catenin signaling pathway and of Notch signaling pathway	none
					
					BCL2*	Integral outer mitochondrial membrane protein that regulate and contribute to programmed cell death or apoptosis	none
					
					CDX2	Transcription factor. Important in broad range of functions from early differentiation to maintenance of the intestinal epithelial lining of both the small and large intestine	none
					
					GATA6	Member of a small family of zinc finger transcription factors that play an important role in the regulation of cellular differentiation and organogenesis during vertebrate development	none
					
					KAT2B*	Functions as a histone acetyltransferase (HAT) to promote transcriptional activation. Inhibits cell-cycle progression	none
					
					PLAG1	Transcription factor whose activation results in up-regulation of target genes, such as IGFII, leading to uncontrolled cell proliferation: when overexpressed in cultured cells, higher proliferation rate and transformation are observed	none

65	1.00	1.00	1.00	miR-25, miR-32,miR-19a	KAT2B*	Functions as a histone acetyltransferase (HAT) to promote transcriptional activation. Inhibits cell-cycle progression	none
					
					PRMT5	Protein arginine methyltransferase 5. Plays a role in the assembly of snRNP core particles. May play a role in cytokine-activated transduction pathways. Negatively regulates cyclin E1 promoter activity and cellular proliferation	none
					
					BCL2L11	Integral outer mitochondrial membrane protein that regulate and contribute to programmed cell death or apoptosis.	none

**Hierarchy level 2**							

16-65	1.00	0.07	0.639	miR-25, miR-32, miR-19a, miR-19b	ESR1^†^	Estrogen receptor. Ligand-activated transcription factor composed of several domains important for hormone binding, DNA binding, and activation of transcription. The steroid hormones and their receptors are involved in the regulation of eukaryotic gene expression and affect cellular proliferation and differentiation in target tissues	Signal Transduction (4.9e-04) Signaling by ERBB4 (1.4e-02)
					
					PTEN*^†^	Phosphatidylinositol-3,4,5-trisphosphate 3-phosphatase. It negatively regulates intracellular levels of phosphatidylinositol-3,4,5- trisphosphate in cells and functions as a tumor suppressor by negatively regulating AKT/PKB signaling pathway	Signal Transduction (4.9e-04) Signaling by SCF-KIT (1.2e-02) Signaling by ERBB4 (1.4e-02) Signalling by NGF (4.6e-02)
					
					ATXN1^†^	Chromatin-binding factor that repress Notch signaling in the absence of Notch intracellular domain by acting as a CBF1 corepressor.	none
					
					BMPR2*^†^	Member of the bone morphogenetic protein (BMP) receptor family of transmembrane serine/threonine kinases. The ligands of this receptor are BMPs, which are members of the TGF-beta superfamily.	Signal Transduction (4.9e-04)
					
					KAT2B*	Functions as a histone acetyltransferase (HAT) to promote transcriptional activation. Inhibits cell-cycle progression.	Signal Transduction (4.9e-04)
					
					PRMT5	Protein arginine methyltransferase 5. Plays a role in the assembly of snRNP core particles. May play a role in cytokine-activated transducion pathways. Negatively regulates cyclin E1 promoter activity and cellular proliferation.	Signal Transduction (4.9e-04)
					
					SOCS1	Suppressor of cytokine signalling. SOCS1 is involved in negative regulation of cytokines that signal through the JAK/STAT3 pathway.	Signaling by SCF-KIT (1.2e-02)
					
					TGFBR2*^†^	Transforming growth factor, beta receptor II. Transmembrane protein that has a protein kinase domain, forms a heterodimeric complex with TGFBR1, and binds TGF-beta. This receptor/ligand complex phosphorylates proteins, which then enter the nucleus and regulate the transcription of a subset of genes related to cell proliferation.	Signal Transduction (4.9e-04)
					
					BCL2L11	Transforming growth factor, beta receptor II. Transmembrane protein that has a protein kinase domain, forms a heterodimeric complex with TGFBR1, and binds TGF-beta. This receptor/ligand complex phosphorylates proteins, which then enter the nucleus and regulate the transcription of a subset of genes related to cell proliferation.	Signalling by NGF (4.6e-02)

Biclusters containing co-targeting on KAT2B. The function of genes is extracted from GeneCards. (*) genes present in bicluster 6-72-22-70. (^†^) new suggested potential targets of miR-25 and miR-32.

Bicluster 41 associates co-targeting of miR-181a and miR-181b on KAT2B with a series of other transcription factors involved in cell fate determination (by different routes like NLK and BCL2) and differentiation (i.e., CDX2, GATA6, PLAG1). This suggests that the cooperation of miR-181a and miR-181b with miR-17-92 may be more specifically related with cell growth and differentiation.

In bicluster 16-65, KAT2B is grouped together with genes which are coordinately regulated by miR-25, miR-32, miR-19a and miR-19b. The enrichment of these genes in pathways (Table 9) suggests that the cooperation of miR-32 with the multiple associations of miR-25 (miR-106b-25 cluster), miR-19a and miR-19b (miR-17-92 cluster) is specifically related to the TGF-β signaling.

Among the biclusters containing genes of miR-17-92, the bicluster 66 is the top ranked (according to GO). Pathway mapping of bicluster 66 returns significant results in the TGF-β/BMP pathway, which regulates embryonic and adult cell proliferation and differentiation, and that is implicated in a great number of human diseases. The transduction of the signal depends on the activation state of different nuclear transcriptional co-activators/co-repressors which can positively or negatively regulate different effectors, so that the interpretation of a signal depends on the cell-type and cross talk with other signaling pathways such as Notch, MAPK and Wnt (^© ^Reactome). Bicluster 66 includes BMPR2 (bone morphogenetic protein receptor type II), TGFBR2 (TGF-β receptor type II) and SMAD4. While BMPR2 and TGFBR2 are key factors for the activation of TGF-β/BMP receptor complexes and for the transduction of the signal from the cell surface to the cytosol, SMAD4 is essential for the transduction to the nucleus for transcriptional regulation (^© ^Reactome). miRNAs grouped in bicluster 66 indicate that the regulation of TGF-β/BMP signaling at nodal check-points of the signal cascade is modulated by the miR-17-92 gene cluster, namely, miR-17, miR-19a, miR-20a and miR-92a. Moreover, as stated before, the presence of BMPR2 and TGFBR2 in the bicluster 16-65 suggests that they may also be functional targets of miR-25 and miR-32. This supports the hypothesis that the activation of the TGF-β receptor is under a complex control mediated by multiple associations of constitutive regulators (e.g. miR-17 and miR-20a), with diverse members of the same cluster, i.e. miR-19a and miR-92, and with miR-25 (miR-106b-25) and miR-32, in a context-specific manner. This also suggests that, among the components of miR-106b-25, miR-25 is the one that contributes to the control of the transmission of the TGF-β signaling from the cell surface to the nucleus.

Genes in biclusters 70 and 72, although different, are enriched in cell cycle regulation. Bicluster 70 shows a significant over-representation of genes in the G1 phase (p-value = 8.0 E-07) and G1/S-phase transition (p-value = 1.9 E-5), whereas bicluster 72 specifically maps in the G1/S-phase transition (p-value = 7.8 E-08). As for miRNAs, biclusters 70 and 72 share miR-17 and miR-20a, but bicluster 70 contains miR-106a and bicluster 72 contains miR-106b. These observations provide useful insights: first, they confirm experimental evidences that demonstrate that miR-17 is a key regulator of cell cycle progression by targeting more than 20 genes involved in the G1/S-phase transition [43]; second, the co-targeting of miR-20a (always associated with miR-17a) underlines that it also cooperate to this pathway-specific role of miR-17. Third, the association of miR-106b in bicluster 70 suggests that miR-106b-25 influences the effects of miR-17-92 in the cell cycle progression by controlling regulatory circuits involving E2F3, RBL1 and RBL2, while the association of miR-106a in bicluster 72 suggests that miR-106a-363 influences the effects of miR-17-92 on cell cycle progression by keeping under control regulatory circuits involving CDKN1, E2F1 and RB1. This last aspect may help to shed light on the role of miR-106a-363 in the general function of the miR-17-92 cluster family [40].

#### Discussion on potential applications of extracted biclusters

A general conclusion of the analysis reported in the previous subsection is that our results match with validated experimental results reported in the current literature, demonstrating that HOCCLUS2 is able to provide valuable clues for the understanding of miRNAs functions and mechanisms. In this subsection, we mainly discuss potential uses of biclusters extracted by HOCCLUS2.

As shown for biclusters 41, 65 and 16-65, neither GO-based ranking nor the analysis of gene enrichment in pathways provide complete understanding on the quality of discovered interaction networks. Nevertheless, we have proved that these biclusters provide important insights for the clarification of functions and interaction networks involving miR-17-92 components. This example clarifies both the usefulness and effectiveness of HOCCLUS2, even when results are not supported by statistical confirmations on existing resources. It is noteworthy that the statistical ranking of target genes in GO depends on the completeness of annotations available and on the gene classification in the GO tree. Thus, although GO ranking is used to score biclusters, it has not to be intended as an exclusive criterion for the evaluation of the quality of biclusters and for the consequent analysis of data. Rather, it has to be considered as an indicator of potential functional correlations which depend on annotation systems and, as such, it could inevitably fail because of poor, wrong or incomplete annotations.

As regards possible applications, results obtained by HOCCLUS2 on miRTarBase can be used to retrieve all the significant multiple interactions that a miRNA (or a set of miRNAs) of interest may have. Performing this task manually on the source database (miRTarBase in this case) would require to execute a large set of queries and to analyze and aggregate tens of thousands of results. Nevertheless, all this effort would not provide any information on significant gene correlations and miRNA context-specific multiple interactions.

Furthermore, HOCCLUS2 (which is freely available) can be easily applied to the analysis of other collections of data, e.g. to the analysis of data obtained in specific physiological and pathological conditions which may greatly contribute to the elucidation of miRNAs functions in the relative context. Similarly, HOCCLUS2 could be applied to the analysis of miRNA:mRNA interactions in other organisms annotated in miRTarBase as well as in organisms and plants for which still poor annotations on validated targets are available. In this last case, combining microarrays data with target predictions would allow the researchers to easily detect potentially significant multiple interactions which are worth to be experimentally validated.

In addition to the possibility to extract multiple and significant co-targeting of miRNAs, HOCCLUS2 is able to give new clues in the identification of still unknown miRNA targets. This possibility is due to its ability to associate objects, by iteratively merging pairs of biclusters, that are apparently not related. By observing the biclusters analyzed in the previous subsection, the bicluster 16-65 appears to be a good candidate for suggesting the validation of still unknown targets of miR-25, miR-32, miR-19a and miR-19b. The cohesiveness value of this bicluster, *q *= 0.639, indicates that around the 64% of all the possible interactions between its miRNAs and mRNAs are in the dataset and, since they come from miRTarBase, are experimentally validated. This means that the hypothesis that the remaining 36% of possible interactions could actually exist is based on the 64% of validated interactions. This observation confirms that the cohesiveness-preserving strategy adopted by HOCCLUS2 is effective, since, intuitively, the higher the cohesiveness of a bicluster, the higher the probability that the discovered (but not present in the database) interactions actually exist. Indeed, a deep analysis of the interactions of bicluster 16-65 revealed that, in miRTarBase, all the genes are validated targets of miR-19a and miR-19b with the exception of PRMT5, which is a validated target of miR-25, miR-32 and miR-19a but not of miR-19b. Moreover, KAT2B and BCL2L11 are validated targets of all the miRNAs in the bicluster. Looking at prediction data in mirDIP, it is possible to find some predictions which support the hypothesis that the remaining interactions actually exist. In particular, at least one algorithm predicted: ESR1, PTEN, ATXN1, BMPR2, KAT2B, TGFBR2 and BCL2L11 as targets of miR-19a, miR-19b, miR-25 and miR-32; PRMT5 as target of miR-19a, miR-25 and miR-32; SOCS1 as target of miR-19a and miR-19b, but not of miR-25 and miR-32. These observations lead to the conclusion that, in addition to KAT2B and BCL2L11, it would be worth to experimentally validate the hypothesis that ESR1, PTEN, ATXN1, BMPR2 and TGFBR2 are targets of miR-25 and miR-32.

#### Comparison of results on miRTarBase with results on mirDIP

Conversely by results of the analysis carried out on miRTarBase, results on biclusters extracted from mirDIP datasets cannot be considered impressive. Although some biclusters have presented enough enrichment in genes that fall in the same or related biological processes and although validated miRNAs interactions have been detected, the much larger number of genes involved has not allowed us a so detailed analysis as for miRTarBase data. On the other hand, Reactome, as other similar resources, still misses pathway mapping annotations for many genes, thus negatively affecting statistical enrichment analysis. In particular, searching for biclusters of the miR-17-92 gene cluster family in mirDIP has led to identify a few biclusters which were not so well defined as those extracted from miRTarBase, even though functional characterization by pathways mapping has returned a picture that well matches with functional properties of miR-17-92.

In the attempt of motivating this different behavior, we have searched for predictions of validated targets of miR-17-92 components in mirDIP. We have found that the difference in the quality of the results obtained on miRTarBase and on mirDIP were mainly due to the performance of prediction algorithms in detecting actual targets. For example, TargetScan Conserved predictions present very low standardized scores for those genes that have been largely confirmed as targets of miR-17-92 (e.g., E2F1, E2F3 and CDKN1A are predicted targets of mir-17 and mir-20a with a standardized score ranging from 0.337 to 0.392; CDKN1A is a predicted target of mir-106b with a standardized score of 0.358).

## Conclusions

In this work, we tackle the problem of biclustering miRNAs and mRNAs on the basis of their interactions. In order to solve this problem, by taking into account specific issues raised by this task, we propose the algorithm HOCCLUS2 which extracts hierarchically organized and overlapping biclusters by maximizing biclusters cohesiveness and exploiting statistical distribution of the data.

The performance of our method is evaluated in terms of execution time and bicluster cohesiveness on a dataset of experimentally verified miRNA:mRNA interactions, i.e. miRTarBase, as well as on miRNA target predictions extracted from mirDIP. A comparative analysis shows that HOCCLUS2 is able to extract a set of (hierarchically organized) biclusters with significantly higher cohesiveness values than ROCC, in a comparable execution time, which proves the inappropriateness of the application of gene expression biclustering algorithms to discover meaningful biclusters from miRNA:mRNA interactions.

The effectiveness of the algorithm in extracting biologically related biclusters is automatically tested and confirmed on the basis of the GO classification. Furthermore, an in-depth biological analysis proves that functional relationships among miRNAs and mRNAs in the same bicluster (at different levels of the hierarchy) find large confirmation in the literature. This indicates that the algorithm is able to extract valuable knowledge and that its application in the biological domain may provide us good insights in the study of complex miRNA mechanisms and functions. Moreover, it also proves that the algorithm could be considered as a valid tool for the detection of candidate new miRNAs target genes.

Current results of HOCCLUS2 on miRTarBase human dataset may already be used to easily map differentially expressed miRNAs from microarrays experiments in miRNA:mRNA interacting modules. On the other hand, the application of HOCCLUS2 on very large datasets of predicted targets of differentially expressed miRNAs, although in some way impaired by the poor effectiveness of the prediction algorithms, may significantly help in suggesting potential significant interactions among the huge amount of results they produce. For future work, we intend to use HOCCLUS2 for multi-label classification purposes, according to the predictive clustering framework [45].

## Availability of supporting data

**Project Name: **HOCCLUS2

**Project Home Page: **http://www.di.uniba.it/~ceci/micFiles/systems/HOCCLUS2/index.html

**Available resources: **HOCCLUS2 software and user manual, all the datasets and all the obtained results.

## Competing interests

The authors declare that they have no competing interests.

## Authors' contributions

MC and GP contributed to the definition of the method. DD contributed to the conception of the biological investigation. GP and MC contributed to design the software. GP and DD took care of the review and selection of bioinformatics resources. GP implemented the system and ran the experiments. DD performed the biological analysis and validation of the results. GP and MC performed the analysis of the results, from the computer science point of view. MC, GP, DD and CL contributed to the manuscript drafting. MC, GP, DD and DM contributed to the manuscript finalization. DM and MC supervised the study. All the authors read and approved the final manuscript.

## Declarations

The work presented in this paper is funded by the FAR project "MBLab: The Molecular Biodiversity LABoratory Initiative".

## Supplementary Material

Additional file 1 **Reactome results on bicluster 6-72-22-70**. Click here for file
