# Supplementary material for: A Novel Biclustering Algorithm for the Discovery of Meaningful Biological Correlations between microRNAs and their Target Genes
Source: BMC Bioinformatics. 2013 Apr 22;14(Suppl 7):S8. doi: 10.1186/1471-2105-14-S7-S8 (PMC3633049; doi:10.1186/1471-2105-14-S7-S8)
Supplement: Additional file 1 — Reactome results on bicluster 6-72-22-70. [file 1471-2105-14-S7-S8-S1.pdf]

Supplementary File 1 - Reactome results on bicluster 6-72-22-70

False discovery rate Un-adjusted probability of seeing N or more genes in this Event by chance
Number of genes in your query which map to this Event Total number of genes involved in this Event
Identifier of this Event Name of this Event Submitted identifiers mapping to this Event
-1.51182628499782e-13 8 38 REACT_821 Cyclin D associated events in G1 E2F1, E2F3, RB1, CDKN1A, RBL2, CCND2, RBL1, CCND1
-1.51182628499782e-13 8 38 REACT_1590 G1 Phase E2F1, E2F3, RB1, CDKN1A, RBL2, CCND2, RBL1, CCND1
-5.64696440441308e-13 6 12 REACT_1882 Cyclin D:Cdk4/6 mediated phosphorylation of Rb and dissociation of phospho-Rb from the E2F1/2/3:DP-1 complexes E2F1, E2F3, RB1, CDKN1A, CCND2, CCND1
-1.47930576111424e-10 9 133 REACT_21267 Mitotic G1-G1/S phases E2F1, E2F3, RB1, CDKN1A, RBL2, CCND2, WEE1, RBL1, CCND1
-2.9423735496367e-08 4 11 REACT_111248 Cyclin D:CDK4/6 mediated phosphorylation of p107 (RBL1) and dissociation of phosphorylated p107 (p-RBL1) from DP1:E2F4 complex CDKN1A, CCND2, RBL1, CCND1
-4.40404344496045e-08 4 12 REACT_111206 Cyclin D:Cdk4/6 mediated phosphorylation of p130 (RBL2) and dissociation of phosphorylated p130 (RBL2) from DP1:E2F4/5 complex CDKN1A, RBL2, CCND2, CCND1
-5.12949722586043e-07 9 336 REACT_152 Cell Cycle, Mitotic E2F1, E2F3, RB1, CDKN1A, RBL2, CCND2, WEE1, RBL1, CCND1
-1.10115755016645e-06 3 7 REACT_2081 Formation of Cyclin D:Cdk4/6 complexes CDKN1A, CCND2, CCND1
-1.10115755016645e-06 3 7 REACT_713 Translocation of Cyclin D:Cdk4/6 complexes from the cytoplasm to the nucleus CDKN1A, CCND2, CCND1
-2.9194067388945e-06 9 413 REACT_115566 Cell Cycle E2F1, E2F3, RB1, CDKN1A, RBL2, CCND2, WEE1, RBL1, CCND1
-3.75121916187681e-06 3 10 REACT_96 Phosphorylation of Cyclin D:Cdk4/6 complexes CDKN1A, CCND2, CCND1
-3.15569649124271e-05 2 3 REACT_1277 Replication initiation regulation by Rb1/E2F1 E2F1, RB1
-6.96257848609192e-05 3 25 REACT_111214 G0 and Early G1 E2F1, RBL2, RBL1
-7.6097762257074e-05 4 71 REACT_6844 Signaling by TGF-beta Receptor Complex TGFBR2, SMAD4, MYC, RBL1
-0.000104768142163583 2 5 REACT_1915 G2 Phase E2F1, E2F3
-0.000104768142163583 2 5 REACT_9021 Association of Cyclin A:phospho-Cdk2(Thr 160) with E2F1/E2F3 E2F1, E2F3
-0.000104768142163583 2 5 REACT_9023 Phosphorylation of E2F1/E2F3 by Cyclin A:phosph-Cdk2(Thr 160) E2F1, E2F3
-0.000134336329969611 3 31 REACT_120734 SMAD2/SMAD3:SMAD4 heterotrimer regulates transcription SMAD4, MYC, RBL1
-0.000156836922282904 2 6 REACT_111167 Transcription of E2F targets under negative control by DREAM complex E2F1, RBL1
-0.000219131228175128 2 7 REACT_9007 Phosphorylation of proteins involved in the G1/S transition by Cyclin A:Cdk2 RB1, CDKN1A
-0.000291588943261686 2 8 REACT_121097 SMAD2/3:SMAD4 heterotrimer forms a complex with RBL1, E2F4/5 and DP1/2 SMAD4, RBL1
-0.000373868722333886 4 107 REACT_1783 G1/S Transition E2F1, RB1, CDKN1A, WEE1
-0.000415537985951919 4 110 REACT_899 S Phase RB1, CDKN1A, WEE1, CCND1
-0.000439331437829174 3 46 REACT_121061 Transcriptional activity of SMAD2/SMAD3:SMAD4 heterotrimer SMAD4, MYC, RBL1
-0.000468282650687512 3 47 REACT_21351 Exocytosis of platelet alpha granule contents THBS1, APP, VEGFA
-0.000681820878771477 2 12 REACT_329 Inhibition of replication initiation of damaged DNA by RB1/E2F1 E2F1, RB1
-0.0011082415994404 3 63 REACT_1574 Cyclin E associated events during G1/S transition RB1, CDKN1A, WEE1
-0.00116026061961749 3 64 REACT_9029 Cyclin A:Cdk2-associated events at S phase entry RB1, CDKN1A, WEE1
-0.00205607129193984 3 78 REACT_318 Platelet degranulation THBS1, APP, VEGFA
-0.00213062767979125 2 21 REACT_75900 Inflammasomes APP, BCL2
-0.00233899850756423 2 22 REACT_2111 Orc1 is phosphorylated by cyclin A/CDK2 RB1, CDKN1A
-0.00245681819007056 3 83 REACT_1280 Response to elevated platelet cytosolic Ca2+ THBS1, APP, VEGFA
-0.00255663246913833 2 23 REACT_12034 Signaling by BMP BMPR2, SMAD4
-0.00350091729171047 3 94 REACT_21391 Mitotic G2-G2/M phases E2F1, E2F3, WEE1
-0.00378172938001817 2 28 REACT_75829 PIP3 activates AKT signaling PTEN, CDKN1A
-0.00378172938001817 2 28 REACT_120850 TGF-beta receptor signaling activates SMADs TGFBR2, SMAD4
-0.00522981652428562 2 33 REACT_471 E2F mediated regulation of DNA replication E2F1, RB1
-0.00654413201176741 2 37 REACT_12464 PI3K/AKT activation PTEN, CDKN1A
-0.00689389676434976 2 38 REACT_115961 PI3K events in ERBB4 signaling PTEN, CDKN1A
-0.0072520269467886 2 39 REACT_12578 GAB1 signalosome PTEN, CDKN1A
-0.00726319960551336 3 122 REACT_16888 Signaling by PDGF THBS1, PTEN, CDKN1A
-0.00916627608545078 2 44 REACT_116008 PI3K events in ERBB2 signaling PTEN, CDKN1A
-0.00970359739943947 11 1664 REACT_111102 Signal Transduction THBS1, PTEN, TGFBR2, VEGFA, CDKN1A, RBL1, KAT2B, APP, BMPR2, SMAD4, MYC
-0.010411788338289 2 47 REACT_118780 NOTCH1 Intracellular Domain Regulates Transcription MYC, KAT2B
-0.0140764282700432 2 55 REACT_75913 Nucleotide-binding domain, leucine rich repeat containing receptor (NLR) signaling pathways APP, BCL2
-0.0150683865673572 2 57 REACT_21270 PI-3K cascade PTEN, CDKN1A
-0.0222179697979647 2 70 REACT_1156 Orc1 removal from chromatin RB1, CDKN1A
-0.0222179697979647 2 70 REACT_2148 Switching of origins to a post-replicative state RB1, CDKN1A

-0.0234217722263485 2 72 REACT_207 Removal of licensing factors from origins RB1, CDKN1A
-0.0234217722263485 2 72 REACT_829 Regulation of DNA replication RB1, CDKN1A
-0.0240336809318218 2 73 REACT_118859 Signaling by NOTCH1 MYC, KAT2B
-0.0271914480413554 2 78 REACT_111040 Signaling by SCF-KIT PTEN, CDKN1A
-0.0291616508914763 3 205 REACT_798 Platelet activation, signaling and aggregation THBS1, APP, VEGFA
-0.03169291268765 4 371 REACT_12627 Generic Transcription Pathway SMAD4, MYC, RBL1, KAT2B
-0.0375996331136461 2 93 REACT_17025 Downstream signal transduction PTEN, CDKN1A
-0.0375996331136461 2 93 REACT_115596 Signaling by ERBB4 PTEN, CDKN1A
-0.0398404653480469 2 96 REACT_2014 Synthesis of DNA RB1, CDKN1A
-0.0413624126593847 2 98 REACT_118638 Downstream Signaling Events Of B Cell Receptor (BCR) PTEN, CDKN1A
-0.0429064192730137 2 100 REACT_21272 Downstream signaling of activated FGFR PTEN, CDKN1A
-0.0444721560361185 2 102 REACT_115755 Signaling by ERBB2 PTEN, CDKN1A
-0.0492965010057483 2 108 REACT_9417 Signaling by EGFR PTEN, CDKN1A
